# Accuracy of Deep Learning for Detecting Axillary Lymph Node Metastasis in Breast Cancer: Systematic Review and Meta-Analysis

**DOI:** 10.2196/77593

**Published:** 2026-04-16

**Authors:** Xueying Wang, Tiantian Li, Xiaohang Wang, Deyuan Fu

**Affiliations:** 1 Department of Breast Surgery, Northern Jiangsu People's Hospital Affiliated to Yangzhou University, No.98 Nantong West Road, Guangling District, Yangzhou, 225001, China, 86 18051060677; 2 Department of Ultrasonography, Northern Jiangsu People's Hospital Affiliated to Yangzhou University, Yangzhou, China; 3 Institute of Translational Medicine, Jiangsu Key Laboratory of Integrated Traditional Chinese and Western Medicine for Prevention and Treatment of Senile Diseases, Medical College, Yangzhou University, Yangzhou, China

**Keywords:** breast cancer, axillary lymph node metastasis, deep learning, systematic review, ultrasound, magnetic resonance imaging, computed tomography

## Abstract

**Background:**

Axillary lymph node metastasis (ALNM) is an important factor in detecting breast cancer (BC). However, the noninvasive diagnosis of ALNM remains challenging. While some deep learning (DL) models have been developed for preoperative ALNM assessment, their performance lacks systematic evaluation.

**Objective:**

This study aims to evaluate the effectiveness of DL in detecting ALNM, providing evidence to support clinical diagnostic tools.

**Methods:**

Embase, Web of Science, PubMed, and Cochrane Library were searched from their inception through January 26, 2026. The Quality Assessment of Diagnostic Accuracy Studies was used to assess the risk of bias in the included studies. A bivariate mixed effects model was applied for analysis, and subgroup analyses were conducted based on different imaging modalities.

**Results:**

This meta-analysis included 28 independent studies and pooled data from 20,811 patients with BC. Among them, 7123 cases had confirmed ALNM. The overall diagnostic performance of the DL model (bivariate mixed effects) for detecting ALNM in BC was as follows: sensitivity 0.80 (95% CI 0.76‐0.84), specificity 0.85 (95% CI 0.80‐0.88), diagnostic odds ratio (DOR) 22 (95% CI 16‐30), and area under the summary receiver operating characteristic curve (AUC) 0.89 (95% CI 0.86‐0.92). The positive likelihood ratio (LR+) was 5.2 (95% CI 4.1‐6.5), and the negative likelihood ratio (LR−) was 0.24 (95% CI 0.19‐0.29). For ultrasound-based DL models targeting ALNM detection, the pooled sensitivity and specificity were 0.79 (95% CI 0.72‐0.84) and 0.86 (95% CI 0.79‐0.91), respectively. Diagnostic performance metrics showed an LR+ of 5.5 (95% CI 3.8‐8.1), an LR− of 0.25 (95% CI 0.19‐0.32), a DOR of 22 (95% CI 15‐33), and an AUC of 0.89 (95% CI 0.86‐0.91). Regarding magnetic resonance imaging–based DL models for detecting ALNM, the pooled sensitivity was 0.78 (95% CI 0.71‐0.83) and the pooled specificity was 0.82 (95% CI 0.76‐0.87). Corresponding metrics included an LR+ of 4.4 (95% CI 3.3‐5.9), an LR− of 0.27 (95% CI 0.21‐0.35), a DOR of 16 (95% CI 11‐25), and an AUC of 0.87 (95% CI 0.84‐0.90). For computed tomography (CT)–based models, the sensitivity was 0.90 (95% CI 0.78‐0.96), the specificity was 0.88 (95% CI 0.84‐0.92), and the AUC was as high as 0.91 (95% CI 0.89‐0.94).

**Conclusions:**

Current DL methods for detecting ALNM in BC primarily utilize ultrasound, magnetic resonance imaging, and CT. DL models based on all 3 modalities demonstrated good diagnostic performance. CT had the highest sensitivity and AUC, while its specificity was comparable to that of ultrasound. These findings provide supportive evidence for the development or optimization of clinical diagnostic models.

## Introduction

Breast cancer (BC) is a common and life-threatening tumor in women. According to the latest global statistics of 2025, there were 2.3 million new BC cases (accounting for 25% of all new cancer cases among women) and 670,000 BC-related deaths (accounting for 15.5% of cancer deaths in women) worldwide in 2022. By 2050, new cases of BC are projected to rise by 38%, while BC deaths are expected to increase by 68%, with the fastest growth observed in low- and middle-income countries [1]. The incidence of BC varies notably by region. New Zealand and Australia have the highest incidence of BC, with an age-standardized incidence rate of 100.3 per 100,000, while South Asia has the lowest rate at 26.7 per 100,000 [1]. Moreover, the incidence of BC is rising at approximately 0.6% per year and is trending toward a younger age at onset [2,3]. Consequently, BC has emerged as a severe disease burden on society. BC treatment includes local and systemic therapies, with surgery, radiotherapy, and chemotherapy as the primary therapeutic approaches. Currently, immunotherapy [4], brachytherapy [5], and neoadjuvant therapy [6] have significantly improved the clinical outcomes and prolonged the survival of patients with BC. However, some patients still experience poor prognoses due to influencing factors, such as axillary lymph node metastasis (ALNM), molecular subtypes, and lymphovascular invasion. As ALNM can elevate the risk of recurrence and metastasis of BC, it is an important factor for assessing the treatment and prognosis of BC [7]. Currently, the accurate detection of ALNM before BC surgery remains challenging. Although techniques including fine-needle aspiration cytology and core needle biopsy can be used in clinical practice, these invasive procedures may lead to complications such as implantation metastasis and hematoma. Moreover, these procedures may cause false negatives, thereby affecting diagnostic accuracy and timely treatment. Thus, developing a preoperative, efficient, accurate, and safe method for detecting ALNM is of great clinical significance for improving the diagnostic and prognostic evaluation of patients with BC. Furthermore, it serves as a critical research topic that requires in-depth exploration.

With advances in machine learning (ML), many researchers have applied ML approaches to oncology. ML can integrate high-dimensional data, including clinical features, gene expression, and imaging characteristics, to construct models for diagnosing disease, assessing progression, and evaluating prognosis [8-10]. Traditional ML depends on manual coding and requires variable selection, image segmentation, and extraction of image features using specialized software (eg, 3D Slicer [Slicer Community] and ITK-SNAP [Penn Image Computing and Science Laboratory]). The features are then imported into ML for filtering, modeling, and validation. However, information may be lost during the extraction and screening process. Although deep learning (DL) can automatically train on images, it still relies on manual segmentation for model construction in current research. DL can directly train models based on segmented images. Therefore, this technique preserves maximal information, enhancing the diagnostic accuracy [11-13]. Accordingly, some researchers have developed image-based DL models [14-16] to improve the preoperative diagnosis of ALNM. Nonetheless, systematic evidence on the accuracy of DL for detecting ALNM remains limited. Therefore, this study aims to evaluate the performance of current image-based DL models for detecting ALNM in BC and provide evidence-based insights for developing or updating intelligent diagnostic tools for clinical practice.

## Methods

### Study Registration

This study was performed in accordance with the PRISMA (Preferred Reporting Items for Systematic Reviews and Meta-Analyses) 2020 guidelines (Checklist 1) and was registered in the PROSPERO (Prospective Register of Systematic Reviews) database (ID CRD42024609828).

### Eligibility Criteria

The eligibility criteria were established as the basis for subsequent literature screening as shown in Table 1.

**Table 1.** Eligibility criteria for study inclusion in the systematic review and meta-analysis of DL^a^ models for detecting ALNM^b^ status in patients with BC^c^.

PICOS	Inclusion criteria	Exclusion criteria
Participants (P)	Studies involving patients with BC	Studies that did not strictly differentiate BC from other tumors
Intervention (I)	Studies that developed DL models for ALNM detection	Studies that only applied traditional ML^d^ Studies that only performed image segmentation without constructing a DL model
Comparison (C)	None	None
Outcomes (O)	Studies that reported metrics for evaluating ML model performance (eg, sensitivity, specificity, area under the summary receiver operating characteristic curve, negative likelihood ratio, positive likelihood ratio, and diagnostic odds ratio)	Studies that did not report the aforementioned performance metrics
Study design (S)	Case-control, cohort, or cross-sectional studies Studies published in English	Conference abstracts, guidelines, meta-analyses, reviews, or expert opinions Lymph node status not confirmed by biopsy No clear differentiation between the sentinel and axillary lymph nodes

a DL: deep learning.

b ALNM: axillary lymph node metastasis.

c BC: breast cancer.

d ML: machine learning.

### Data Sources and Search Strategy

Web of Science, Cochrane Library, PubMed, and Embase were searched from their inception up to January 26, 2026. The search strategy was designed by combining free-text terms and subject headings. There were no temporal or geographical restrictions. The search strategy is illustrated in Table S1 in Multimedia Appendix 1.

### Study Selection and Data Extraction

Initially retrieved studies were imported into EndNote. After removing duplicates, the titles and abstracts of the remaining studies were further reviewed. After a full-text review, eligible studies were selected. Data extraction was performed using a standardized form. The extracted information included digital object identifier, publication year, patient source, country, title, study design, first author, number of ALNM cases, total cases, number of ALNM cases within the training cohort, total cases within the training cohort, number of ALNM cases within the validation cohort, total cases within the validation cohort, methods used to generate the validation cohort, whether region of interest segmentation was performed, model type, and whether a clinical comparison was conducted. Two reviewers independently selected the studies and extracted the data. Disagreements were resolved through consultation with a third reviewer.

### Risk of Bias in Included Studies

The risk of bias (RoB) of the included studies was assessed using the Quality Assessment of Diagnostic Accuracy Studies [17]. This tool assessed overall bias and clinical applicability in 4 domains: index test, flow and timing, reference standard, and patient selection. Each domain was rated as low, high, or unclear RoB according to specific criteria. Two reviewers independently assessed the RoB and cross-checked the evaluation results. Disagreements were resolved through consultation with a third reviewer.

### Synthesis Methods

Data analysis was performed using Stata 15.0 (StataCorp LLC). A bivariate mixed effects (BME) model was used to explore the nonlinear relationship between sensitivity and specificity. The model estimated the following along with their corresponding 95% CIs: pooled sensitivity, specificity, area under the summary receiver operating characteristic curve (AUC), negative likelihood ratio (LR−), positive likelihood ratio (LR+), and diagnostic odds ratio (DOR). Publication bias was evaluated using the Deeks funnel plot. Sensitivity and specificity were derived from a 2×2 contingency table. Since most studies did not report contingency tables, the pooled sensitivity, specificity, precision, and case numbers were used for calculations. In the validation phase, a meta-analysis was conducted. When multiple validation sets were available within a single investigation, all were incorporated into the analysis. Subgroup analyses were performed, stratified by image type and validation set generation method. Furthermore, the analysis aggregated the outcomes from both internal and external validations for DL models developed using ultrasound, magnetic resonance imaging (MRI), and computed tomography (CT). Moreover, for studies with multiple validation cohorts, only the cohort with the highest Youden index was retained to ensure that a single validation cohort was retained in each study for the sensitivity analysis. The meta-analysis was then repeated with this reduced dataset. A *P* value <.05 was considered statistically significant.

## Results

### Study Selection

**Figure 1.**
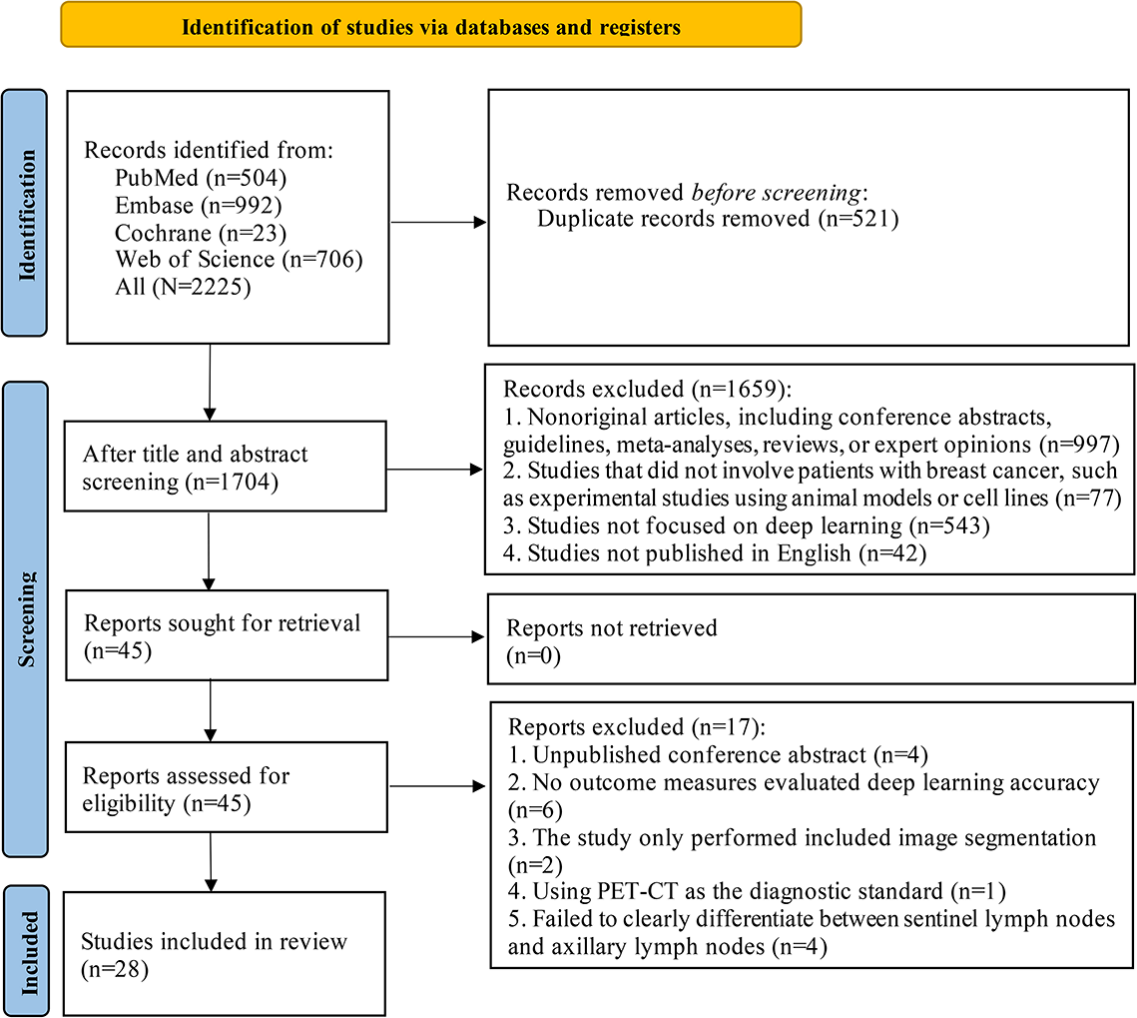
PRISMA (Preferred Reporting Items for Systematic Reviews and Meta-Analyses) flow diagram illustrating the identification and selection process of studies evaluating DL models for the preoperative diagnosis of ALNM in BC. Data sources: Web of Science, Cochrane Library, PubMed, and Embase (search date: January 26, 2026). ALNM: axillary lymph node metastasis; BC: breast cancer; PET-CT: positron emission tomography-computed tomography.

A total of 2225 studies were selected from the databases, among which 521 duplicates were removed. Following a title and abstract screening, 1659 studies were further excluded. The full texts of the remaining 45 studies were assessed, and 17 studies were removed for the following reasons: inaccessible conference abstracts (n=4), absence of outcome indicators for assessing the accuracy of DL (n=6), sole focus on image segmentation (n=2), use of positron emission tomography-CT as the diagnostic gold standard (n=1), and lack of clear differentiation between sentinel and axillary lymph nodes (n=4). Ultimately, 28 studies [14,16,18-43] were included in the analysis. The study screening process is illustrated in Figure 1.

### Study Characteristics

A total of 28 studies published between 2018 and 2026 were included. These studies involved 20,811 patients with BC, 7123 of whom had ALNM. Twenty-three of the studies were from China, 3 from the United States, 1 from Italy, and 1 from Korea. All of the studies used a case-control design. Seventeen of the studies were single-center, and 11 were multicenter. Regarding imaging modalities, 10 studies utilized MRI, 14 used ultrasound images, and 4 used CT scans. All 28 studies clearly described their validation methods. Twenty studies employed random sampling, 10 used external validation, and 3 utilized cross-validation. Twenty-three studies conducted manual segmentation, and 7 reported the comparisons between DL models and clinical physicians (Table 2).

**Table 2.** Characteristics of the 28 included studies on DL^a^ for ALNM^b^ in patients with BC^c^ published between 2018 and 2026^d^.

No.	Included study and year of publication	Country of the author	Study type	Patient source	Modality	Number of lymph node metastasis cases	Total number of cases	Total number of cases in the training set	Generation method of the validation set	Number of cases in the validation set	Whether region of interest segmentation is used or not	Segmentation method	Network architecture	Network input	Comparison with clinicians
1	Gong et al [18] (2025)	China	Case-control	Single center	US^e^	312	1280	921	Random sampling, temporal validation	I1^f^: 102; I2: 257	Yes	Manual	Transformer	2D	No
2	Agyekum et al [19] (2025)	China	Case-control	Multicenter	US	295	820	621	External validation	E1^g^: 112; E2: 87	Yes	Manual	ResNet50^h^ and GCN^i^	2D	No
3	Li et al [20] (2025)	China	Case-control	Multicenter	US	1013	1557	1144	Random sampling, external validation	I: 273; E: 140	Yes	Manual	Transformer	2D	No
4	Gu et al [21] (2025)	China	Case-control	Multicenter	MRI^j^	201	520	287	Random sampling, external validation	I: 124; E: 109	Yes	Semiautomatic	Convolutional neural network (CNN)	3D	No
5	Dai et al [22] (2025)	China	Case-control	Multicenter	MRI	386	935	742	Random sampling	I: 83; E: 110	Yes	Manual	3D ResNet, 3D-Xception, and HRNet^k^	2D	Yes
6	Sun et al [23] (2025)	China	Case-control	Single center	CT^l^	91	258	Not reported	Random sampling	Not reported	Yes	Manual	ResNet101	2D	No
7	Wang et al [24] (2024)	China	Case-control	Single center	US	67	266	212	Random sampling	I: 27; T: 27(3)	Yes	Automatic	DeepLabV3+, ResNet-101, and CNN	2D	No
8	Polat et al [25] (2024)	America	Case-control	Single center	MRI	152	253	All cases	Cross-validation (2)	All cases	Yes	Manual	CNN	3D	No
9	Zhou et al [26] (2024)	China	Case-control	Multicenter	MRI	530	1259	801	Random sampling	I: 344; E: 114	Yes	Manual	ResNet101, ResNeXt101, and DenseNet^m^	2D	Yes
10	Park et al [14] (2024)	Korea	Case-control	Single center	CT	303	523	417	Random sampling	I1: 53; I2: 53	Yes	Manual	DenseNet 121	2D axial CT slice	No
11	Liu et al [27] (2024)	China	Case-control	Multicenter	US	327	883	621	External validation	E1: 112; E2: 87; E3: 63	Yes	Manual	ResNet50	2D US image	Yes
12	Guo et al [28] (2024)	China	Case-control	Multicenter	MRI	1064	2063	1256	Random sampling, external validation	I: 539; E1: 153; E2: 115	Yes	Manual	Convolutional recurrent neural network	2D image	No
13	Wei et al [29] (2023)	China	Case-control	Single center	US	349	892	535	Random sampling	I1: 178; I2: 179	Yes	Manual	Inception_v3, ResNet101, ResNet50, VGG19^n^, and Wide_resnet50_v2	2D grayscale image	No
14	Li et al [16] (2023)	China	Case-control	Multicenter	US	127	320	210	External validation	I: 61; E: 49	Yes	Automatic	CNN (R2+1D, TIN^o^, ResNet-3D)	Video sequence (3D)	Yes
15	Gao et al [30] (2023)	China	Case-control	Multicenter	MRI	387	941	742	Random sampling, external validation	I: 83; E: 116	Yes	Manual	3D ResNet + CBAM^p^	3D DCE^q^-MRI volume data	Yes
16	Zhang et al [31] (2022)	China	Case-control	Single center	MRI	98	252	202	Random sampling	50	Yes	Manual	ResNet50	2D	No
17	Zhang et al [32] (2022)	China	Case-control	Single center	US	394	952	902	External validation	50	No	None	CNN	2D	Yes
18	Wang et al [33] (2022)	China	Case-control	Single center	MRI	163	348	315	Random sampling	33	Yes	Manual	ResNet50	2D	No
19	Sun et al [34] (2022)	America	Case-control	Single center	US	64	169	All cases	Cross-validation	All cases	Yes	Manual	Custom CNN and ResNet-101	2D	No
20	Santucci et al [35] (2022)	Italy	Case-control	Single center	MRI	27	128	All cases	Cross-validation	All cases	Yes	Manual	SFB-NET^r^, VB-NET^s^, 2DS-NET^t^ (optimal)	2D slice and 3D volume	No
21	Li et al [36] (2022)	China	Case-control	Single center	US	489	2131	1491	Random sampling	640	Yes	Manual	ResNet-50	2D	No
22	Cattell et al [37] (2022)	America	Case-control	Single center	MRI	67	198	109	Random sampling	I1: 54; I2: 35	Yes	Manual	VGG16	2D	No
23	Zeng et al [38] (2021)	China	Case-control	Single center	CT	100	229	153	Random sampling	76	Yes	Manual	Custom CNN (decoupling convolution)	2D	No
24	Liu et al [39] (2021)	China	Case-control	Single center	CT	400	800	480	Random sampling	I1: 160; I2:160	Yes	Manual	DA-VGG19	2D	No
25	Zhou et al [40] (2020)	China	Case-control	Multicenter	US	420	834	680	Random sampling, external validation	I: 76; E: 78	No	None	Inception V3, Inception-ResNet V2, and ResNet-101	2D	Yes
26	Zheng et al [41] (2020)	China	Case-control	Single center	US	247	584	466	Random sampling	118	Yes	Manual	ResNet-50	2D	No
27	Sun et al [42] (2020)	China	Case-control	Single center	US	136	479	359	Random sampling	120	Yes	Manual	DenseNet-121	2D	No
28	Guo et al [43] (2020)	China	Case-control	Multicenter	US	365	937	542	External validation	395	Yes	Manual	Custom CNN	2D	No

a DL: deep learning.

b ALNM: axillary lymph node metastasis.

c BC: breast cancer.

d The table details study design, patient source, imaging modality, and validation methods. The 8th, 19th, and 20th studies in the table employed cross-validation methodologies. In the 3 studies, all cases were used in both the training and validation sets, and the training and validation sets were not independently partitioned. The construction of 2×2 contingency tables was not possible due to missing data, and some study cohorts were not included in the statistical analysis.

e US: ultrasound.

f I: internal validation cohort.

g E: external validation cohort.

h ResNet50: Residual Network 50.

i GCN: graph convolutional network.

j MRI: magnetic resonance imaging.

k HRNet: high-resolution network.

l CT: computed tomography.

m DenseNet: densely connected convolutional network.

n VGG: Visual Geometry Group.

o TIN: temporal interlacing network.

p CBAM: convolutional block attention module.

q DCE: dynamic contrast-enhanced.

r SFB-NET: single fixed-size box network.

s VB-NET: variable-size box network.

t 2DS-NET: 2-dimensional slice network.

### RoB in Included Studies

#### Risk of Selection Bias and Outcome Assessment

All studies adopted consecutive sampling and applied image-based DL. Although these studies followed a case-control design, there was no bias in evaluating the performance of image-based DL. Therefore, the risk of selection bias was considered low as the case exclusions were reasonable. None of the studies described how the gold standard was interpreted. However, since this meta-analysis focused on artificial intelligence (AI)–based DL, the interpretation of the reference standard had minimal impact on the role of DL in assessing positive events. Thus, the RoB in outcome assessment was considered low. The time interval between the reference standard and the index test was appropriate in all 28 studies, each of which used a single, consistent gold standard. Regarding patient flow, all studies were rated as having a low RoB.

#### Concerns Regarding Applicability (High RoB)

However, 3 studies were rated as having a high RoB in the assessment of clinical applicability because they relied solely on cross-validation.

#### Summary of Assessments

**Figure 2.**
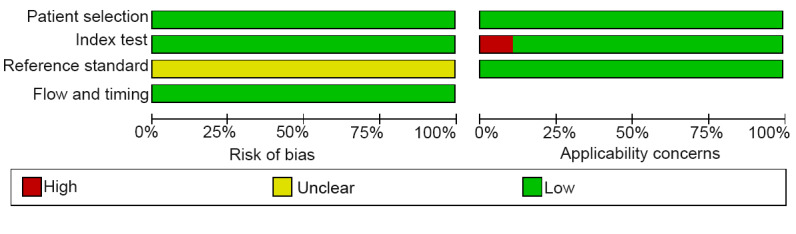
Summary of RoB, as assessed via the QUADAS-2 tool, for the 28 included case-control studies (2018‐2026) that evaluated the image-based DL models in detecting ALNM among patients with BC. The assessment across the 4 domains (patient selection, index test, reference standard, and flow and timing) indicates an overall low RoB. ALNM: axillary lymph node metastasis; BC: breast cancer; DL: deep learning; RoB: risk of bias; QUADAS: Quality Assessment of Diagnostic Accuracy Studies.

The RoB assessments are illustrated in Figures2  3.

**Figure 3.**
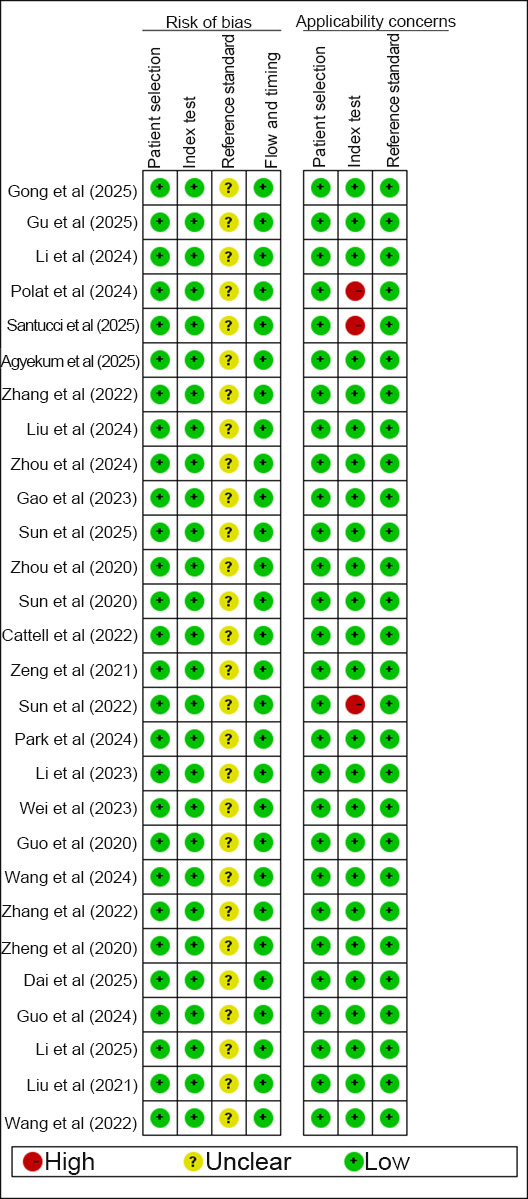
Overall low concerns regarding applicability identified by QUADAS-2 assessment of DL studies for ALNM diagnosis [14,16,18-24,26-33,36-43]. Among the 28 studies evaluated, 3 studies that used only cross-validation raised concerns due to their limited generalizability to broader clinical practice. ALNM: axillary lymph node metastasis; DL: deep learning; QUADAS: Quality Assessment of Diagnostic Accuracy Studies.

#### Overall Model

**Figure 4.**
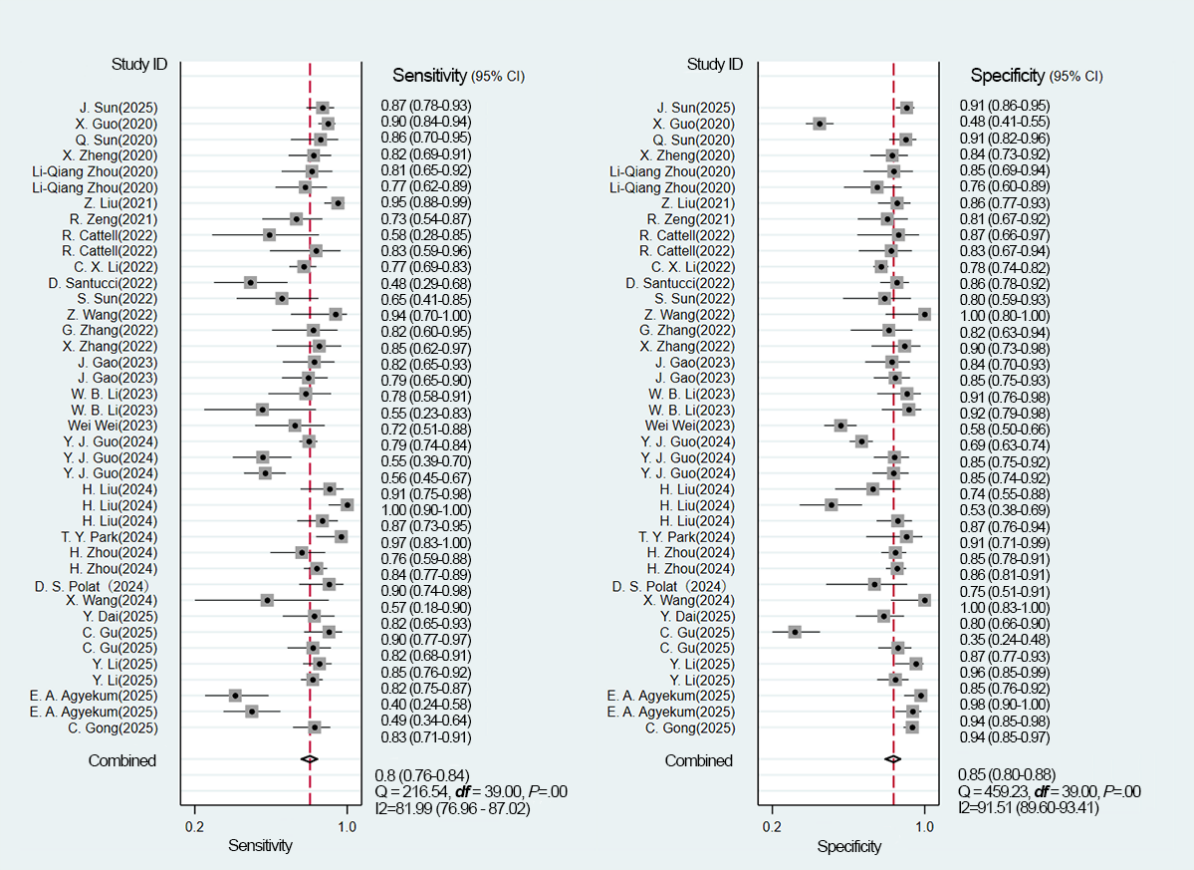
Forest plots showing the pooled sensitivity and specificity of DL models for diagnosing ALNM [14,16,18-43]. The data were pooled from 40 validation cohorts across 28 studies, demonstrating an overall sensitivity of 0.80 (95% CI 0.76‐0.84) and a specificity of 0.85 (95% CI 0.80‐0.88). ALNM: axillary lymph node metastasis; DL: deep learning.

Across all included studies, a total of 40 2×2 contingency tables from validation cohorts were utilized to evaluate the accuracy of DL in detecting ALNM, with an ALNM prevalence of 40.1%. The pooled results from the BME model were as follows: a sensitivity of 0.80 (95% CI 0.76‐0.84), a specificity of 0.85 (95% CI 0.80‐0.88), an LR+ of 5.2 (95% CI 4.1‐6.5), an LR− of 0.24 (95% CI 0.19‐0.29), a DOR of 22 (95% CI 16‐30), and the AUC of 0.89 (95% CI 0.86‐0.92) (Figures4  5). The Deeks funnel plot revealed no significant publication bias among the studies (*P*=.11) (Figure 6). Assuming a prior probability of 0.4, a positive test result corresponded to a true positive (TP) probability of 0.78 (Figure 7).

The internal validation analysis used 26 diagnostic 4-fold tables to evaluate the accuracy of a DL model in detecting ALNM. The observed prevalence of ALNM was 39%. The BME model yielded the following pooled estimates: a sensitivity of 0.82 (95% CI 0.78‐0.85), a specificity of 0.85 (95% CI 0.82‐0.88), an LR+ of 5.6 (95% CI 4.4‐7.0), and an LR− of 0.21 (95% CI 0.18‐0.26). The DOR was 26 (95% CI 18‐38), with an AUC of 0.90 (95% CI 0.87‐0.92) (Figures S1 and S2 in Multimedia Appendix 1). The Deeks funnel plot indicated no significant publication bias (*P*=.21) (Figure S3 in Multimedia Appendix 1). Assuming a prior probability of 0.4, a positive test result corresponded to a TP probability of 0.79 (Figure S4 in Multimedia Appendix 1).

For the external validation, 14 diagnostic 4-fold tables were assessed, with an ALNM prevalence of 43%. The BME model demonstrated a pooled sensitivity of 0.78 (95% CI 0.66‐0.86), a specificity of 0.82 (95% CI 0.72‐0.90), an LR+ of 4.4 (95% CI 2.9‐6.8), an LR− of 0.27 (95% CI 0.19‐0.39), a DOR of 16 (95% CI 10‐26), and an AUC of 0.87 (95% CI 0.84‐0.90; Figures S5 and S6 in Multimedia Appendix 1). The Deeks funnel plot indicated no significant publication bias (*P*=.16; Figure S7 in Multimedia Appendix 1). Assuming a prior probability of 0.4, a positive test result corresponded to a TP probability of 0.75 (Figure S8 in Multimedia Appendix 1).

**Figure 5.**
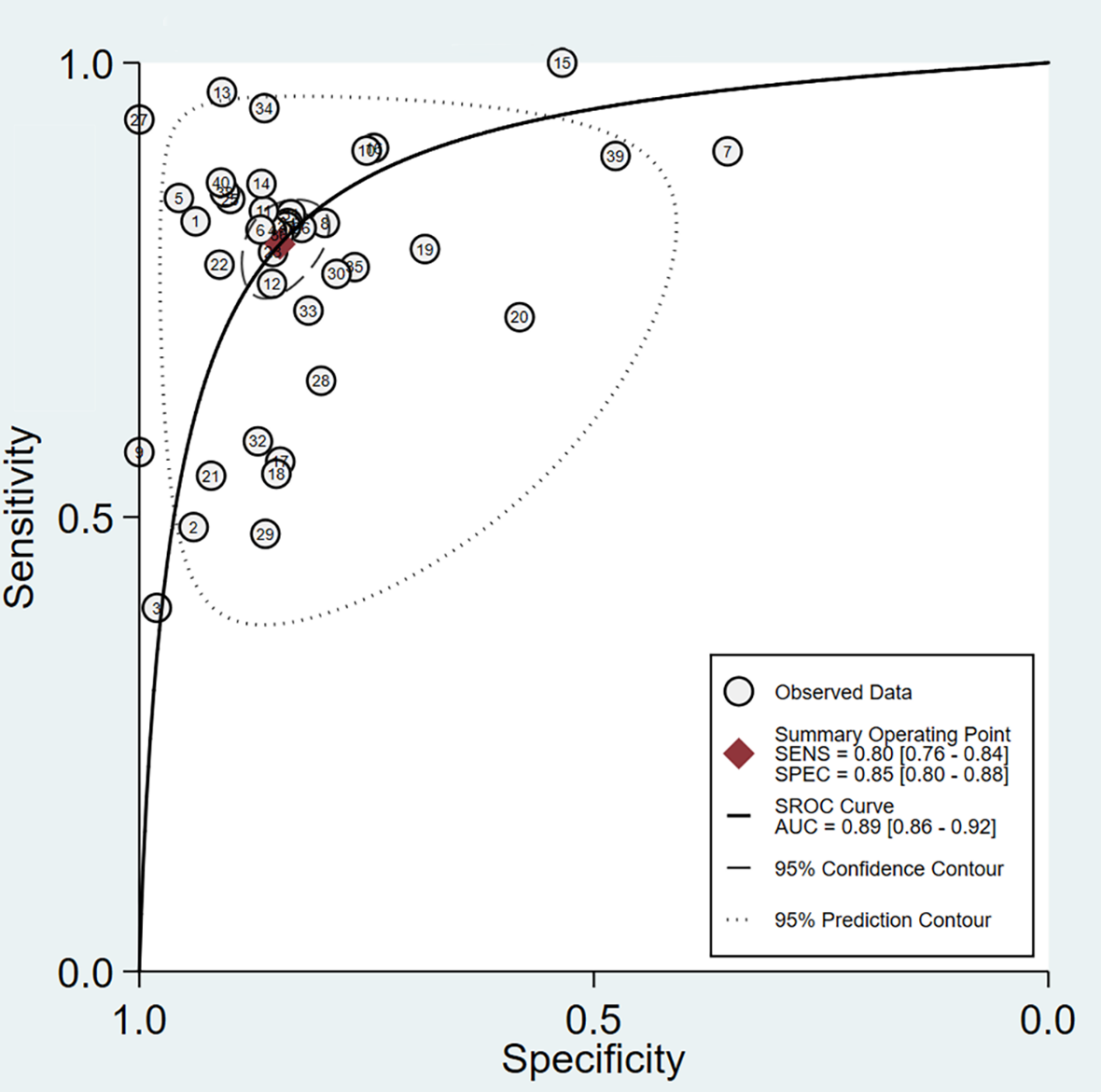
Summary receiver operating characteristic curve (with prediction and confidence contours) from the bivariate meta-analysis evaluating the diagnostic accuracy of DL models for detecting ALNM in BC. The curve is based on 40 contingency tables derived from the validation cohorts of the included studies. The analysis yielded a summary AUC of 0.89 (95% CI 0.86‐0.92). ALNM: axillary lymph node metastasis; AUC: area under the summary receiver operating characteristic curve; BC: breast cancer; DL: deep learning.

**Figure 6.**
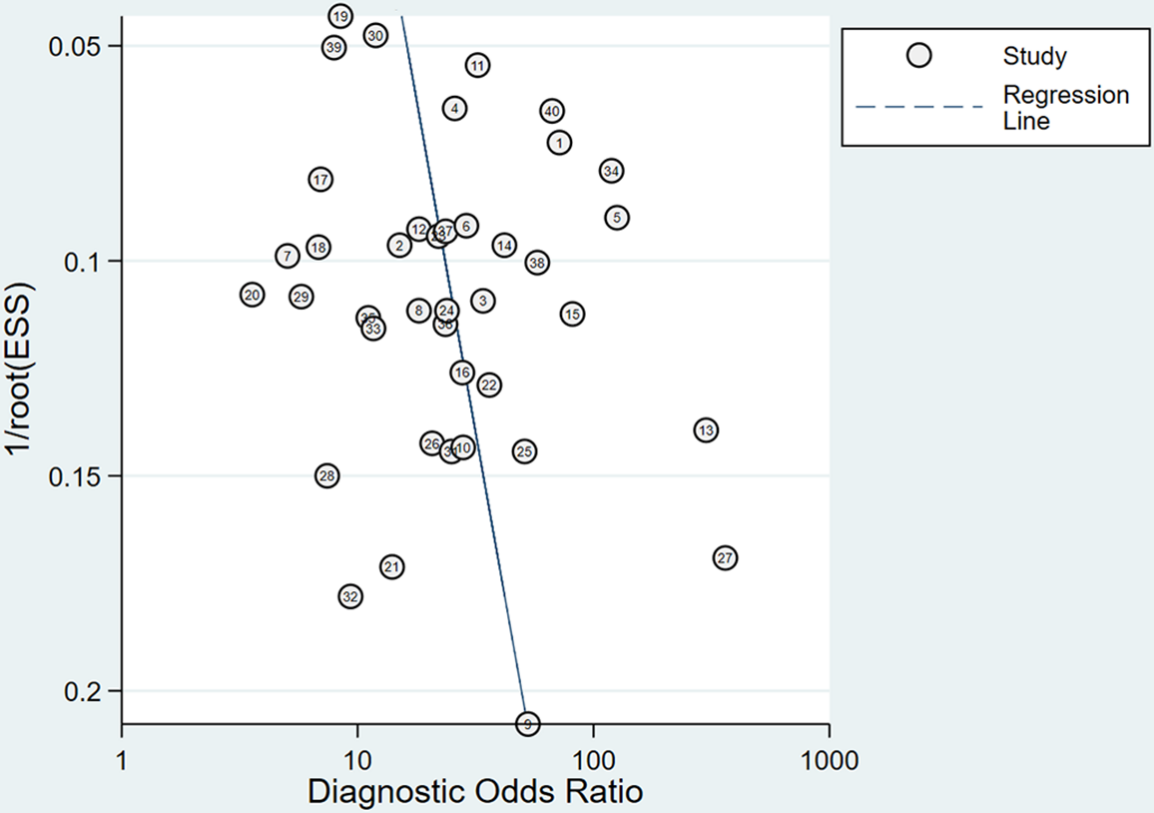
Assessment of potential publication bias using the Deeks funnel plot asymmetry test for the included studies on DL models in detecting ALNM in BC. The test indicated potential publication bias (*P*=.11). ALNM: axillary lymph node metastasis; BC: breast cancer; DL: deep learning; ESS: effective sample size.

**Figure 7.**
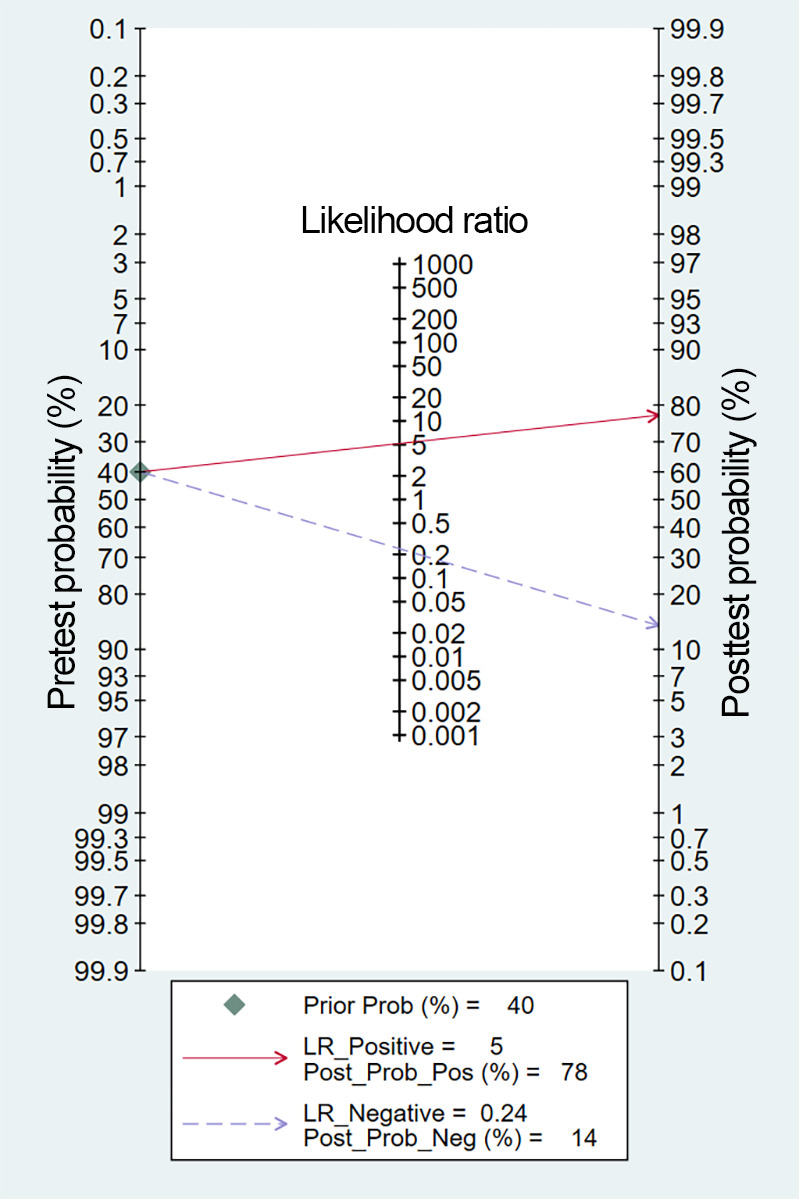
Fagan nomogram illustrating the 78% posttest probability of ALNM following a positive DL model result, given a 40% pretest probability. ALNM: axillary lymph node metastasis; DL: deep learning; LR: likelihood ratio.

#### Conventional Ultrasound

Fourteen studies utilizing conventional ultrasound were included, which provided 20 distinct validation cohorts (due to multiple cohorts from several studies) for assessing DL models, with an ALNM prevalence of 38 %. The pooled results from the BME model were as follows: a sensitivity of 0.79 (95% CI 0.72‐0.84), a specificity of 0.86 (95% CI 0.79‐0.91), an LR+ of 5.5 (95% CI 3.8‐8.1), an LR− of 0.25 (95% CI 0.19‐0.32), a DOR of 22 (95% CI 15‐33), and an AUC of 0.89 (95% CI 0.86‐0.91) (Figures S9 and S10 in Multimedia Appendix 1). The Deeks funnel plot demonstrated no significant publication bias (*P*=.22) (Figure S11 in Multimedia Appendix 1). Assuming a prior probability of 0.4, a positive test result corresponded to a TP probability of 0.79 (Figure S12 in Multimedia Appendix 1).

The internal validation involved 11 diagnostic 4-fold tables to evaluate the accuracy of a DL model in detecting ALNM. The observed ALNM prevalence was 33%. The BME model yielded the following pooled estimates: a sensitivity of 0.80 (95% CI 0.76‐0.83), a specificity of 0.85 (95% CI 0.79‐0.90), an LR+ of 5.5 (95% CI 3.6‐8.3), an LR− of 0.24 (95% CI 0.19‐0.29), a DOR of 23 (95% CI 13‐41), and an AUC of 0.85 (95% CI 0.81‐0.88) (Figures S13 and S14 in Multimedia Appendix 1). The Deeks funnel plot indicated no significant publication bias (*P*=.71) (Figure S15 in Multimedia Appendix 1). Assuming a prior probability of 0.4, a positive test result corresponded to a TP probability of 0.78 (Figure S16 in Multimedia Appendix 1).

For the external validation, 9 diagnostic 4-fold tables were analyzed, with an ALNM prevalence of 47%. The BME model demonstrated a pooled sensitivity of 0.80 (95% CI 0.65‐0.90), a specificity of 0.85 (95% CI 0.71‐0.93), an LR+ of 5.4 (95% CI 2.9‐10.3), an LR− of 0.23 (95% CI 0.13‐0.40), a DOR of 23 (95% CI 13‐44), and an AUC of 0.90 (95% CI 0.87‐0.92; Figures S17 and S18 in Multimedia Appendix 1). The Deeks funnel plot indicated no significant publication bias (*P*=.16; Figure S19 in Multimedia Appendix 1). Assuming a prior probability of 0.4, a positive test result corresponded to a TP probability of 0.78 (Figure S20 in Multimedia Appendix 1).

#### Magnetic Resonance Imaging

Ten studies evaluated DL models based on MRI, with an ALNM prevalence of 42%. The pooled results from the BME model were as follows: a sensitivity of 0.78 (95% CI 0.71‐0.83), a specificity of 0.82 (95% CI 0.76‐0.87), an LR+ of 4.4 (95% CI 3.3‐5.9), an LR− of 0.27 (95% CI 0.21‐0.35), a DOR of 16 (95% CI 11‐25), and an AUC of 0.87 (95% CI 0.84‐0.90; Figures S21 and S22 in Multimedia Appendix 1). The Deeks funnel plot demonstrated no significant publication bias (*P*=.22; Figure S23 in Multimedia Appendix 1). Assuming a prior probability of 0.4, a positive test result corresponded to a TP probability of 0.75 (Figure S24 in Multimedia Appendix 1).

The internal validation encompassed 11 diagnostic 4-fold tables to examine the accuracy of a DL model in detecting ALNM. The observed prevalence of ALNM was 44%. The BME model yielded the following pooled estimates: a sensitivity of 0.80 (95% CI 0.74‐0.86), a specificity of 0.84 (95% CI 0.79‐0.88), an LR+ of 4.9 (95% CI 3.7‐6.6), an LR− of 0.23 (95% CI 0.17‐0.32), a DOR of 21 (95% CI 13‐35), and an AUC of 0.89 (95% CI 0.86‐0.92; Figures S25 and S26 in Multimedia Appendix 1). The Deeks funnel plot indicated no significant publication bias (*P*=.16; Figure S27 in Multimedia Appendix 1). Assuming a prior probability of 0.4, a positive test result corresponded to a TP probability of 0.77 (Figure S28 in Multimedia Appendix 1).

The external validation cohorts for MRI consisted of 4 studies encompassing 5 distinct cohorts, with an ALNM prevalence of 37%. The pooled results were as follows: a sensitivity of 0.73 (95% CI 0.57‐0.85), a specificity of 0.78 (95% CI 0.61‐0.90), an LR+ of 3.4 (95% CI 2.0‐5.8), an LR− of 0.35 (95% CI 0.23‐0.52), a DOR of 10 (95% CI 6‐17), and an AUC of 0.82 (95% CI 0.78‐0.85; Figures S29 and S30 in Multimedia Appendix 1). The Deeks funnel plot indicated no significant publication bias (*P*=.91; Figure S31 in Multimedia Appendix 1). Assuming a prior probability of 0.4, a positive test result corresponded to a TP probability of 0.69 (Figure S32 in Multimedia Appendix 1).

#### Computed Tomography

Regarding CT-based approaches, 4 studies developed DL models utilizing CT images. Of these, 3 studies were conducted in China and 1 in Korea. All 4 studies employed internal validation to generate their validation cohorts, with an ALNM prevalence of 43%. The pooled results were as follows: a sensitivity of 0.90 (95% CI 0.78‐0.96), a specificity of 0.88 (95% CI 0.84‐0.92), an LR+ of 7.8 (95% CI 5.3‐11.5), an LR− of 0.11 (95% CI 0.05‐0.26), a DOR of 68 (95% CI 24‐196), and an AUC of 0.91 (95% CI 0.89‐0.94; Figures S33 and S34 in Multimedia Appendix 1). The Deeks funnel plot indicated no significant publication bias (*P*=.97; Figure S35 in Multimedia Appendix 1). Assuming a prior probability of 0.4, a positive test result corresponded to a TP probability of 0.84 (Figure S36 in Multimedia Appendix 1).

### Chinese Population

Fifteen studies, comprising 34 diagnostic 4-fold tables, validated DL models constructed using Chinese populations, with an ALNM prevalence of 40%. The pooled results from the BME model were as follows: a sensitivity of 0.80 (95% CI 0.76‐0.84), a specificity of 0.85 (95% CI 0.80‐0.88), an LR+ of 5.3 (95% CI 4.1‐6.8), an LR− of 0.23 (95% CI 0.19‐0.28), a DOR of 23 (95% CI 16‐31), and an AUC of 0.89 (95% CI 0.86‐0.92; Figures S37 and S38 in Multimedia Appendix 1). The Deeks funnel plot revealed no significant publication bias (*P*=.08; Figure S39 in Multimedia Appendix 1). Assuming a prior probability of 0.4, a positive test result corresponded to a TP probability of 0.78 (Figure S40 in Multimedia Appendix 1).

### Population of Other Countries

Six studies, comprising 5 diagnostic 4-fold tables, validated DL models constructed using non-Chinese populations, with an ALNM prevalence of 38%. The pooled results from the BME model were as follows: a sensitivity of 0.79 (95% CI 0.59‐0.90), a specificity of 0.84 (95% CI 0.78‐0.89), an LR+ of 5.0 (95% CI 3.5‐7.3), an LR− of 0.25 (95% CI 0.12‐0.53), a DOR of 20 (95% CI 7‐53), and the AUC of 0.85 (95% CI 0.82‐0.88; Figures S41 and S42 in Multimedia Appendix 1). The Deeks funnel plot demonstrated no significant publication bias (*P*=.67; Figure S43 in Multimedia Appendix 1). Assuming a prior probability of 0.4, a positive test result corresponded to a TP probability of 0.77 (Figure S44 in Multimedia Appendix 1).

### Sensitivity Analysis

A sensitivity analysis was conducted by retaining only 1 validation cohort per study. For studies with multiple cohorts, the validation cohort with the highest Youden index was selected.

#### Analysis Based on Conventional Ultrasound Studies

Fourteen studies utilizing conventional ultrasound were included in the assessment of DL models, with an ALNM prevalence of 34%. The pooled results from the BME model were as follows: a sensitivity of 0.79 (95% CI 0.73‐0.84), a specificity of 0.86 (95% CI 0.78‐0.92), an LR+ of 5.8 (95% CI 3.7‐9.1), an LR− of 0.24 (95% CI 0.19‐0.31), a DOR of 24 (95% CI 14‐41), and an AUC of 0.88 (95% CI 0.85‐0.91; Figures S45 and S46 in Multimedia Appendix 1). The Deeks funnel plot demonstrated no significant publication bias (*P*=.24; Figure S47 in Multimedia Appendix 1). Assuming a prior probability of 0.4, a positive test result corresponded to a TP probability of 0.79 (Figure S48 in Multimedia Appendix 1).

#### Analysis Based on MRI Studies

Ten studies evaluated DL models based on MRI, with an ALNM prevalence of 44%. The pooled results from the BME model were as follows: a sensitivity of 0.82 (95% CI 0.75‐0.87), a specificity of 0.84 (95% CI 0.78‐0.88), an LR+ of 5.0 (95% CI 3.6‐6.8), an LR− of 0.22 (95% CI 0.16‐0.30), a DOR of 22 (95% CI 13‐38), and an AUC of 0.90 (95% CI 0.87‐0.92; Figures S49 and S50 in Multimedia Appendix 1). The Deeks funnel plot demonstrated no significant publication bias (*P*=.11; Figure S51 in Multimedia Appendix 1). Assuming a prior probability of 0.4, a positive test result corresponded to a TP probability of 0.77 (Figure S52 in Multimedia Appendix 1).

#### Human Clinical Experts

Seven studies compared the diagnostic performance of human clinical experts in assessing the ALNM status based on imaging. Among them, 4 studies included 7 clinicians who differentiated ALNM using ultrasound. The pooled sensitivity was 0.65 (95% CI 0.59‐0.71), specificity was 0.75 (95% CI 0.70‐0.79), LR+ was 2.6 (95% CI 2.1‐3.2), LR− was 0.46 (95% CI 0.39‐0.56), DOR was 6 (95% CI 4‐8), and the AUC was 0.76 (95% CI 0.20‐0.97; Figures S53 and S54 in Multimedia Appendix 1). The Deeks funnel plot showed no significant publication bias (*P*=.80; Figure S55 in Multimedia Appendix 1). Assuming a prior probability of 0.4, a positive test result corresponded to a TP probability of 0.63 (Figure S56 in Multimedia Appendix 1). Three investigations assessed the performance of human clinicians in diagnosing ALNM via MRI. In the study by Jing Gao et al [30], 3 radiologists independently evaluated MRI scans for ALNM in the validation set, achieving sensitivities between 64.7% and 75.0% and specificities ranging from 70.6% to 83.8%. With the assistance of the attention-based DL model RCNet, diagnostic performance was markedly improved, resulting in sensitivities of 76.5% to 83.3% and specificities of 80.9% to 89.7%. In the study by Y. Dai et al, 3 human experts evaluated the diagnostic ability of MRI for lymph node metastasis in BC (a sample size of 110). The sensitivity ranged from 0.681 to 0.767, and the specificity ranged from 0.746 to 0.841. Furthermore, the study by Zhou et al [26] found that 3 clinical experts achieved sensitivities ranging from 0.660 to 0.745 and specificities from 0.731 to 0.836 in detecting ALNM. When combined with DL, sensitivity increased to 0.787‐0.829 and specificity increased to 0.806‐0.895. These findings underscore the clinical utility of AI as an auxiliary diagnostic tool.

## Discussion

### Main Findings of This Study

This meta-analysis revealed that DL approaches for detecting ALNM in BC primarily relied on MRI, ultrasound, and CT. The sensitivity and specificity for ultrasound-based DL models were 0.79 and 0.86, respectively. Meanwhile, the sensitivity and specificity of MRI-based models were 0.78 and 0.82, respectively. Furthermore, CT-based models demonstrated a sensitivity of 0.90 and a specificity of 0.88. The sensitivity of CT was superior to that of MRI and ultrasound, while no substantial differences in specificity were observed among the 3 modalities. These robust performance metrics validated the potential of DL as a complementary tool to conventional imaging assessments.

### Comparison With Previous Reviews

Currently, ML has gained widespread attention in the diagnosis and treatment of BC. Previous systematic reviews have evaluated the accuracy of ML models based on various imaging modalities. For instance, a meta-analysis by Jing Zhang et al [44], which included 13 studies involving 1618 patients, demonstrated that dynamic contrast-enhanced MRI radiomics had promising diagnostic performance in detecting ALNM and sentinel lymph node metastasis in patients with BC. However, only 2 of these studies employed DL methods, and the pooled sensitivity and specificity were 0.84 (95% CI 0.53‐0.96) and 0.65 (95% CI 0.31‐0.89), respectively. The limited number of studies constrained the interpretation of the results. Another meta-analysis by Chen et al [45], which included 14 studies, focused on ML-based MRI for diagnosing ALNM in patients with BC, and the pooled sensitivity and specificity were 0.79 (95% CI 0.74‐0.84) and 0.77 (95% CI 0.73‐0.81), respectively. This study primarily summarized models, such as support vector machines, logistic regression, and linear discriminant analysis, without specifically addressing the diagnostic accuracy of DL techniques. Eldaly et al [46] systematically reviewed radiomics approaches for detecting ALNM. Their results revealed that the AUC of the included studies varied from 0.72 to 0.93. However, they did not provide a detailed summary of diagnostic performance, thus limiting the assessment of its true accuracy. Liu et al [47] investigated the feasibility of AI algorithms based on CT and MRI for detecting ALNM in BC. They found that MRI-based methods demonstrated a sensitivity of 0.85 (95% CI 0.79‐0.90) and specificity of 0.81 (95% CI 0.66‐0.83), while CT-based methods showed a sensitivity of 0.88 (95% CI: 0.79‐0.94) and specificity of 0.80 (95%CI: 0.69‐0.88). A radiomics study [48] included MRI, ultrasound, CT, and X-ray mammography. A meta-analysis of 30 studies involving a total of 5611 patients was conducted. The pooled sensitivity and specificity of radiomics for detecting ALNM were 0.86 (95% CI 0.82‐0.88) and 0.79 (95% CI 0.73‐0.84), respectively, demonstrating strong overall diagnostic accuracy. Gong et al [48] comprehensively evaluated the performance of radiomics in detecting ALNM. Their results demonstrated an overall diagnostic accuracy of 23 (95% CI 16‐33) and a sensitivity of 0.86 (95% CI 0.82‐0.88). Radiomics serves as a promising noninvasive approach that can contribute to offering new quantitative modalities for disease diagnosis. However, their study did not strictly differentiate between types of ML, and different ML methods demonstrated varying performance in detecting positive events. Furthermore, there was no strict distinction between datasets in their study, with notable variations observed between the results in the training and validation sets.

With the advancement of AI, there has been a shift from traditional ML to DL. DL can analyze complex and diverse data, enabling personalized diagnosis and treatment that aligns with the current trend of precision medicine. Previous meta-analyses have lacked comprehensive evidence on DL models for detecting ALNM in patients with BC across various imaging modalities.

The present meta-analysis only included DL-based studies to minimize the variability of outcomes caused by different modalities. DL offers significant advantages over traditional ML with more advanced techniques for image processing, contributing to the development of diagnostic models for diseases [37]. Our findings suggested that image-based DL for detecting ALNM in BC demonstrated favorable diagnostic performance. MRI, ultrasound, and CT are commonly used techniques for detecting ALNM in BC. However, MRI is not necessary or routinely used in BC diagnosis due to its higher cost. Our findings reveal that CT-based models achieved higher sensitivity, specificity, and AUC than models based on ultrasound and MRI. However, since only 4 studies on CT were included, the results should be validated using larger datasets. Therefore, future research should develop more CT-based diagnostic models using AI to expand its role in diagnosing BC.

The trade-off between sensitivity and specificity for ultrasound and MRI highlights their complementary roles in clinical practice. The high sensitivity of ultrasound models effectively reduces false negatives by excluding metastasis, whereas the high specificity of MRI models improves confirmation of positive cases by minimizing false positives. These findings support the development of an integrated multimodal DL strategy, with ultrasound as an initial screening tool and MRI for confirming difficult cases. However, real-world validation is required.

Before developing DL-based intelligent diagnostic tools, several challenges require attention. First, the impact of different imaging protocols and preprocessing methods on DL performance must be thoroughly examined. Second, DL models typically require large image datasets. Therefore, future studies should incorporate more extensive imaging data to ensure the robustness of the DL model. Third, the impact of population heterogeneity on model performance should be carefully considered. Future research should include multicenter, multiethnic cohorts when developing DL models. Fourth, external validation is essential to enhance the credibility and generalizability of these DL models. Nevertheless, most included studies relied solely on internal validation, making it difficult to conduct external validation across multiple centers. Among the 28 studies, the majority employed single-center internal validation. Therefore, future research should incorporate more multicenter studies to confirm the real-world applicability of these models.

### Study Limitations

This is the first study to systematically investigate the feasibility of DL for detecting ALNM in BC. However, several limitations should be noted. First, despite a comprehensive literature search, the number of included studies was limited, restricting in-depth analysis and the generalizability of the findings. Second, most included studies relied on random sampling validation and lacked diverse external validation, undermining the overall reliability and generalizability of the results. Third, only a few studies directly compared DL models with clinical experts, and the available data were too limited to support a robust effect size analysis. As a result, it remains uncertain whether DL surpasses clinical experts in diagnostic performance. Fourth, many studies did not provide detailed descriptions of critical aspects, such as image acquisition protocols, segmentation techniques, network architectures and inputs, and reference standards (including biopsy methods and reference intervals). Fifth, this meta-analysis relied solely on data reported in the published articles and their supplementary materials. We did not contact the corresponding authors regarding missing or incomplete data. This may have resulted in the exclusion of some potentially eligible studies, which could introduce bias if the missing data differ systematically from the included data. Sixth, most included studies were based on retrospective evidence. Therefore, future research should focus on standardizing image processing methods and conducting prospective, multicenter external validations to develop DL models with enhanced robustness and broader applicability. Finally, this meta-analysis incorporated validation cohorts from the included studies. For studies that provided multiple validation cohorts, each cohort was included as an independent data point in the analysis. This approach may violate the inherent assumption of independence in the standard BME model. While we conducted a sensitivity analysis by extracting only a single validation cohort per original study, this alternative method may also introduce a certain degree of publication bias. Consequently, although DL models show promise for detecting ALNM, the current findings should be interpreted with caution. Future prospective studies with preregistered protocols are needed to verify these results and minimize publication bias.

### Conclusions

This study revealed that DL models based on medical imaging showed promising accuracy in detecting ALNM in patients with BC. These findings may provide an evidence-based foundation for developing intelligent diagnostic tools. However, the findings were primarily based on models developed from single-center, retrospective datasets. Therefore, future studies should adopt a series of strategies, including but not limited to expanding the sample size, integrating multimodal imaging for joint modeling, and conducting prospective validation, to further enhance the performance of DL models while ensuring their applicability and clinical utility.

## Supplementary material

10.2196/77593 Multimedia Appendix 1 The complete electronic search strategies and all supplementary figures and tables for the systematic review. It includes the detailed search syntax for the PubMed, Web of Science, Embase, and Cochrane Library databases (Table S1), as well as 56 supplementary figures (Figures S1-S56) that present the supplementary data, analyses, and visualizations supporting the main findings of the review, such as sensitivity analyses, specificity analyses, the summary receiver operating characteristic curve, Deeks funnel plot, and Fagan nomogram.

10.2196/77593 Checklist 1 PRISMA checklist.

## Abbreviations

AI: artificial intelligence
ALNM: axillary lymph node metastasis
AUC: area under the summary receiver operating characteristic curve
BC: breast cancer
BME: bivariate mixed effects
CT: computed tomography
DL: deep learning
DOR: diagnostic odds ratio
LR+: positive likelihood ratio
LR−: negative likelihood ratio
ML: machine learning
MRI: magnetic resonance imaging
PRISMA: Preferred Reporting Items for Systematic Reviews and Meta-Analyses
PROSPERO: Prospective Register of Systematic Reviews
RoB: risk of bias
TP: true positive
