# Supplementary material for: Accuracy of Deep Learning for Detecting Axillary Lymph Node Metastasis in Breast Cancer: Systematic Review and Meta-Analysis
Source: J Med Internet Res. 2026 Apr 16;28:e77593. doi: 10.2196/77593 (PMC13085980; doi:10.2196/77593)
Supplement: Multimedia Appendix 1 [file jmir-v28-e77593-s001.doc]

## Multimedia Appendix

**
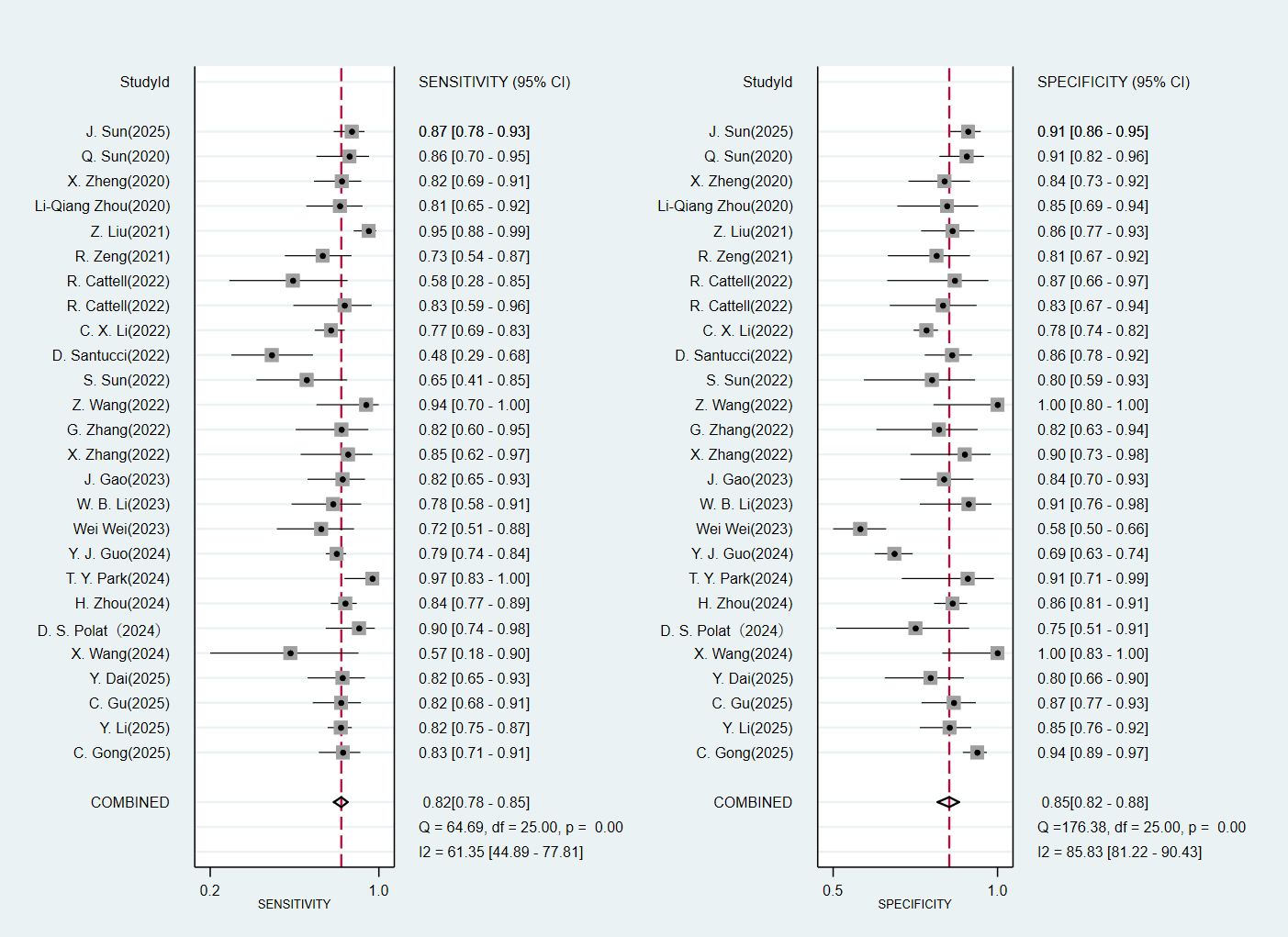
**

**Figure S1** For the internal validation, Forest plots showing the pooled sensitivity and specificity of DL models for diagnosing ALNM

**
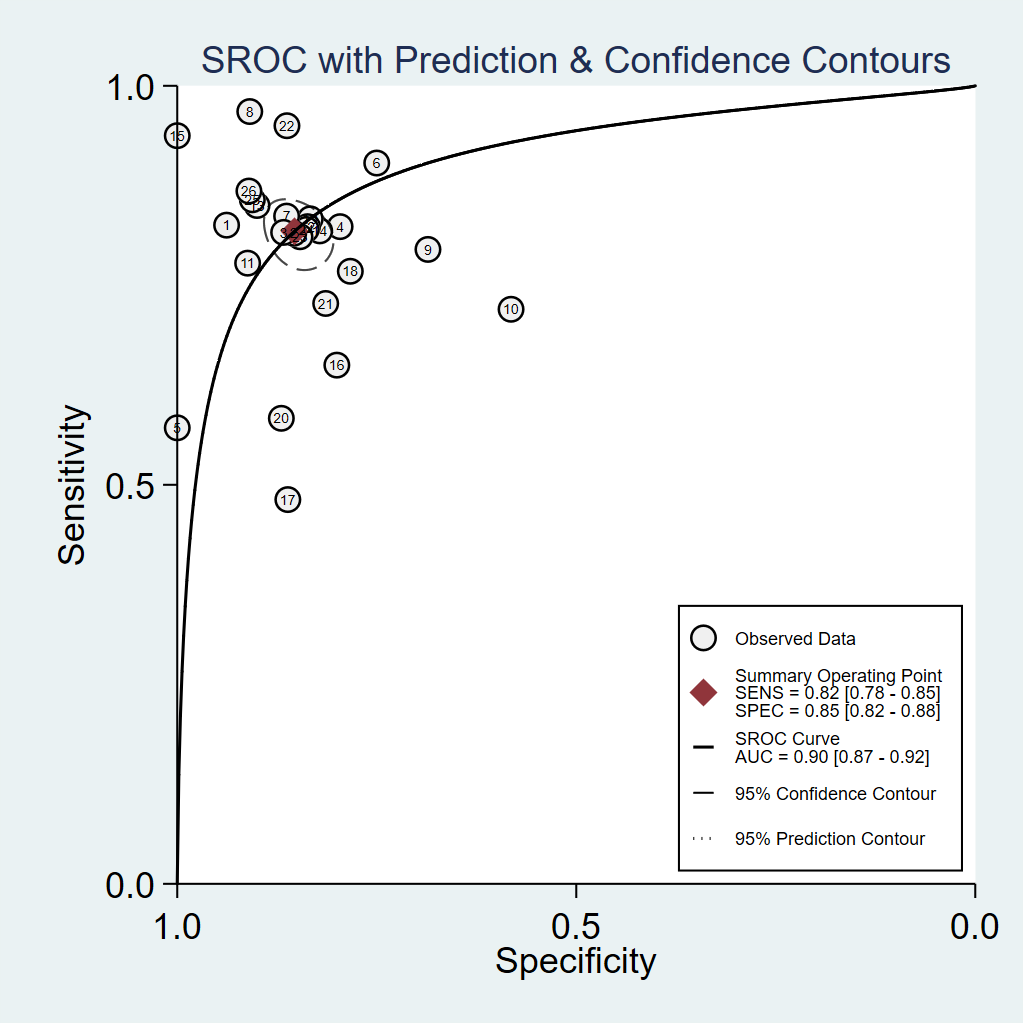
**

**Figure S2** For the internal validation, SROC curve from the bivariate meta-analysis evaluating the diagnostic accuracy of DL models for detecting ALNM in BC

**
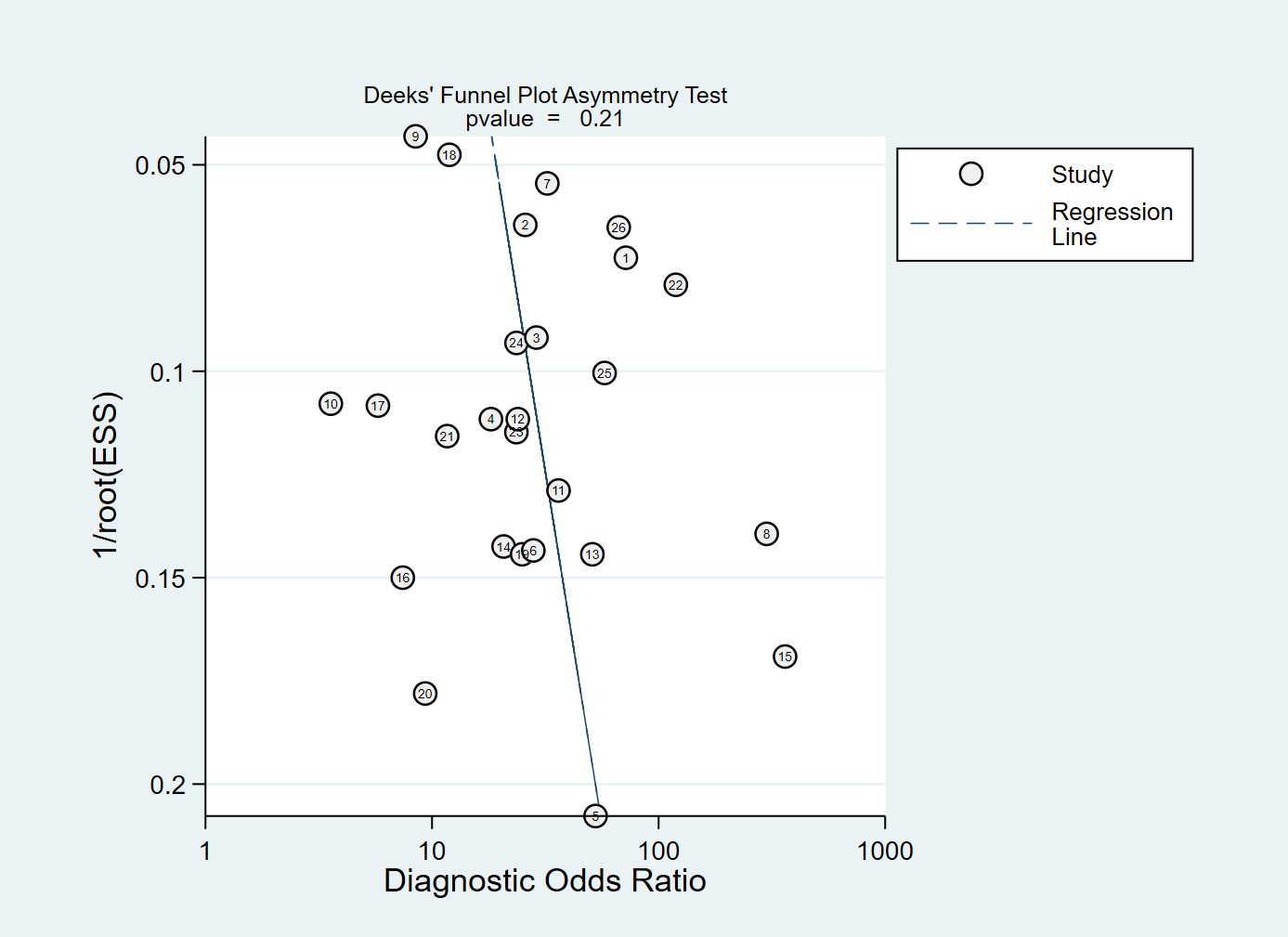
**

**Figure S3** For the internal validation, Assessment of potential publication bias using Deeks' funnel plot asymmetry test for the included studies on DL models in detecting ALNM in BC

**
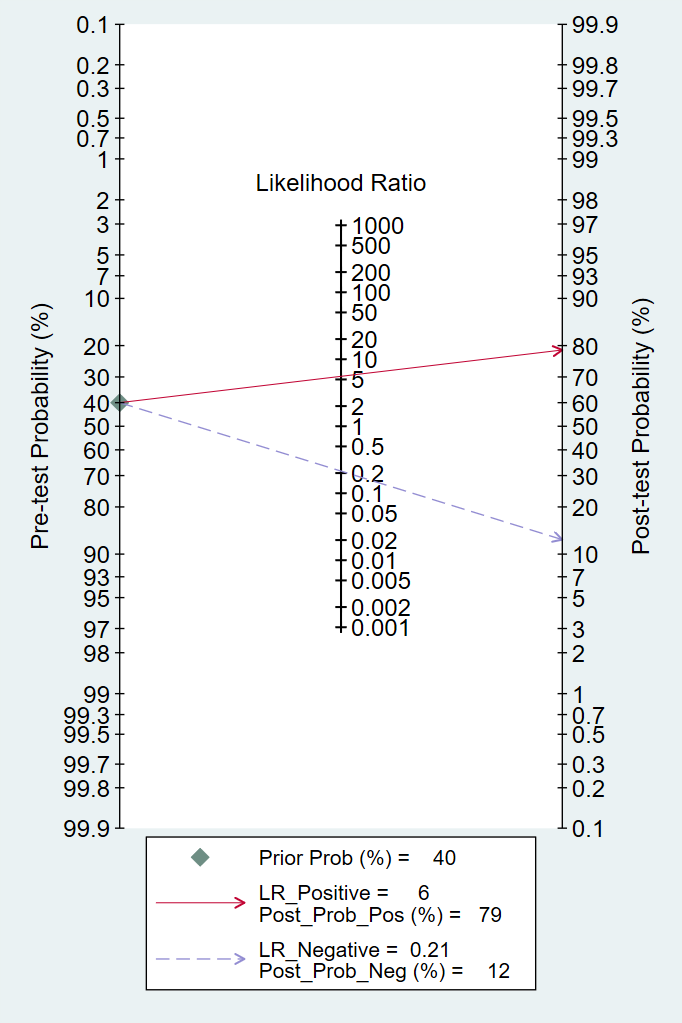
**

**Figure S4** For the internal validation, Fagan's nomogram illustrating the 79% post-test probability of ALNM following a positive DL model result, given a 40% pre-test probability


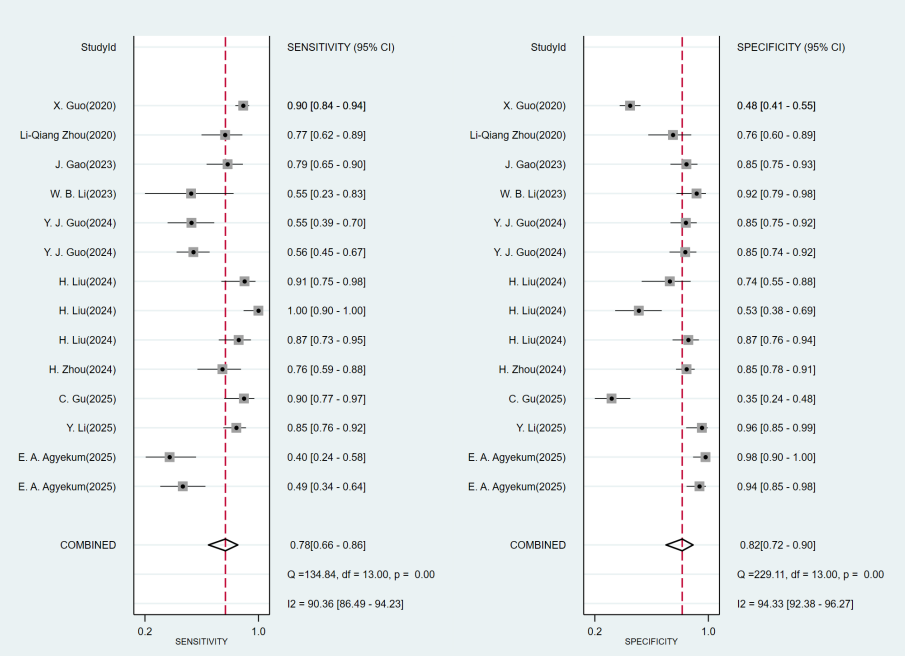


**Figure S5** For the external validation, Forest plots showing the pooled sensitivity and specificity of DL models for diagnosing ALNM


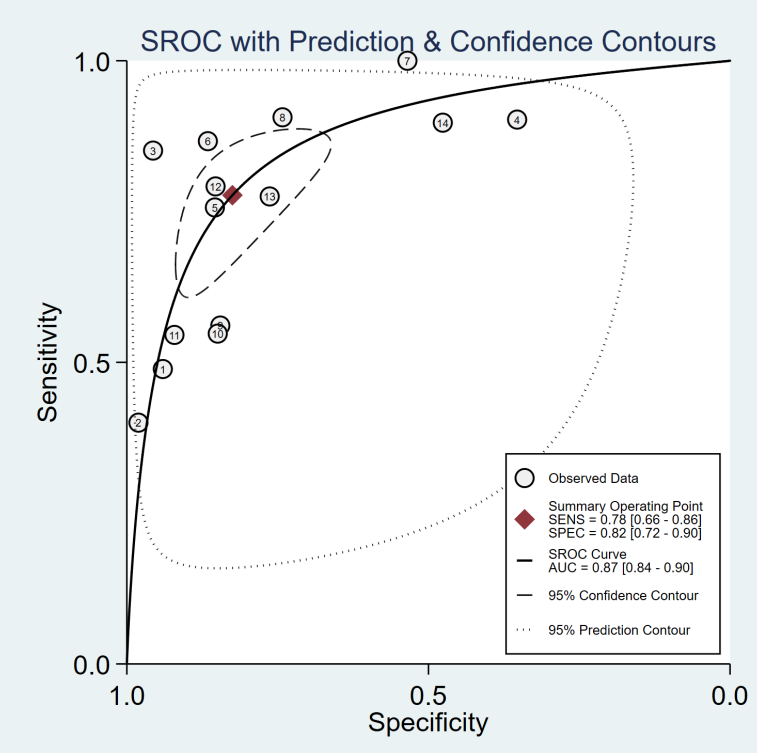


**Figure S6** For the external validation, SROC curve from the bivariate meta-analysis evaluating the diagnostic accuracy of DL models for detecting ALNM in BC


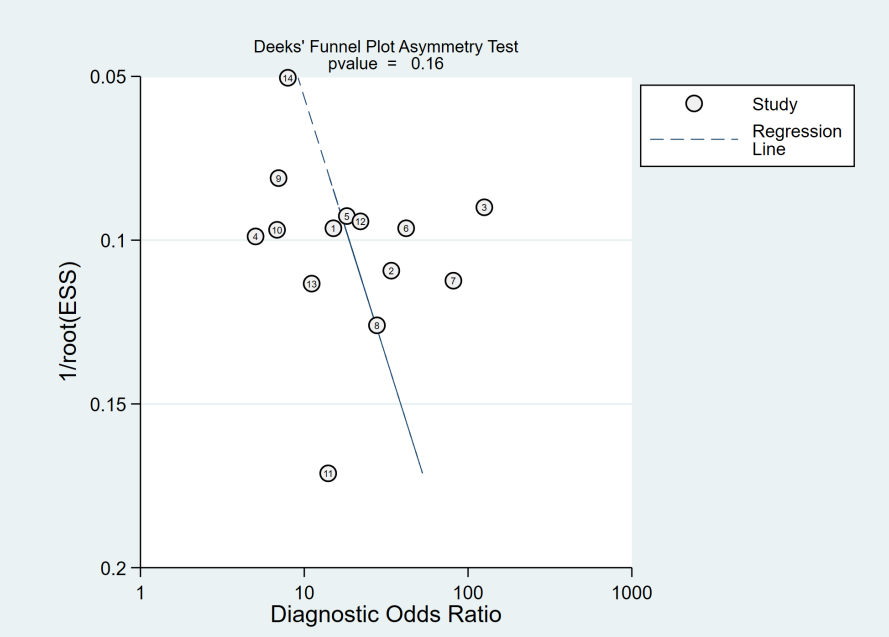


**Figure S7** For the external validation, Assessment of potential publication bias using Deeks' funnel plot asymmetry test for the included studies on DL models in detecting ALNM in BC


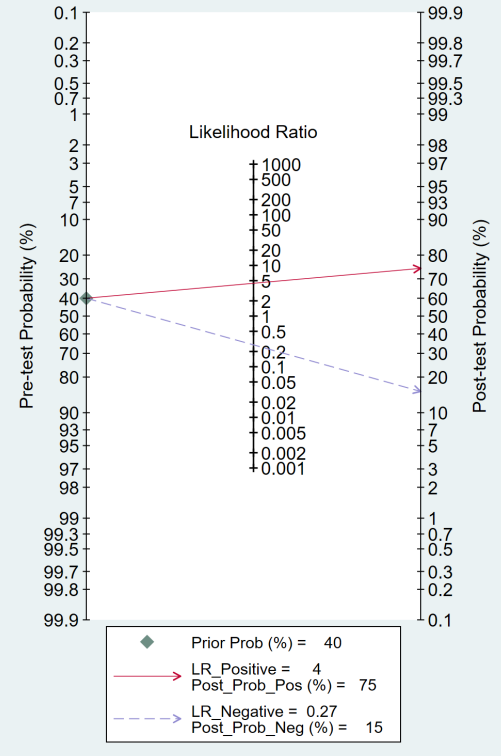


**Figure S8** For the external validation, Fagan's nomogram illustrating the 75% post-test probability of ALNM following a positive DL model result, given a 40% pre-test probability


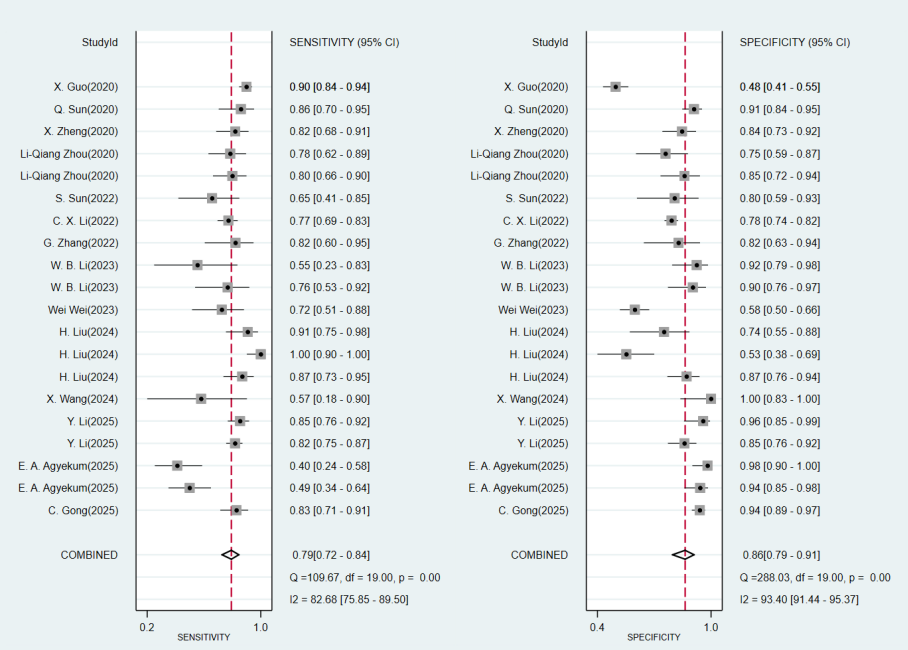


**Figure S9** Forest plot of sensitivity and specificity for DL in detecting ALNM based on conventional US


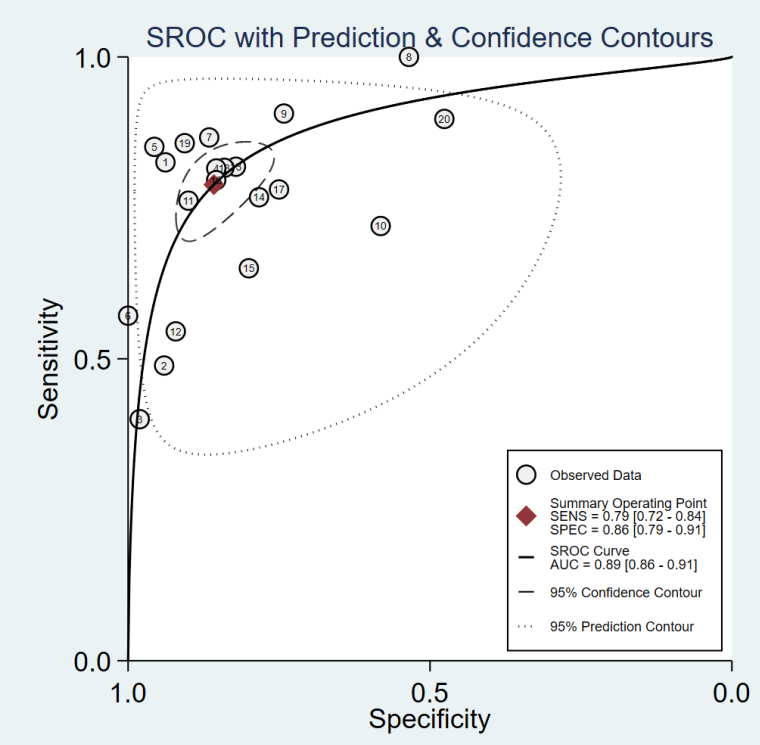


**Figure S10** SROC curve for DL in detecting ALNM based on conventional US


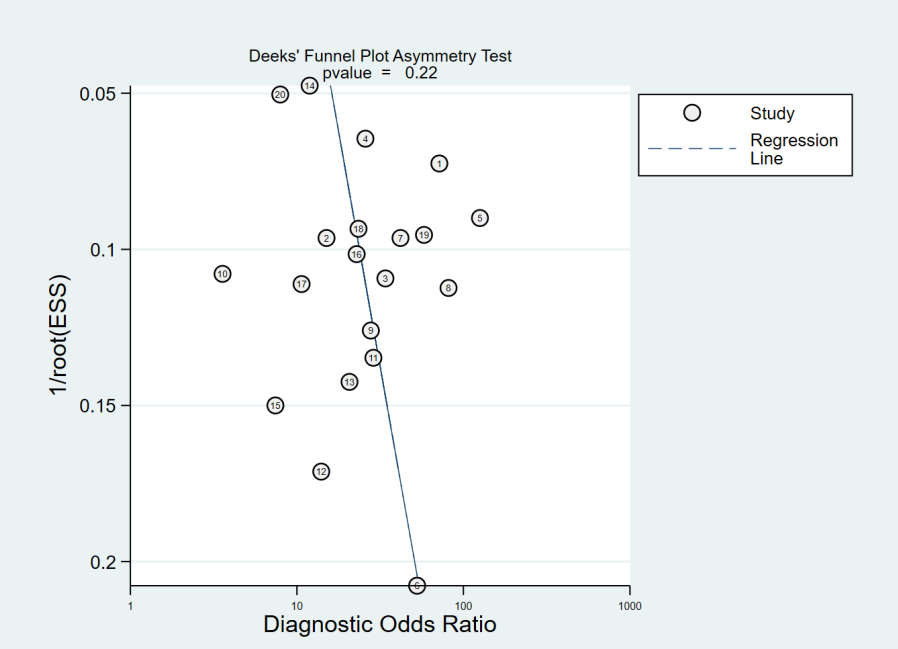


**Figure** **S11** Deeks' funnel plot for DL in detecting ALNM based on conventional US


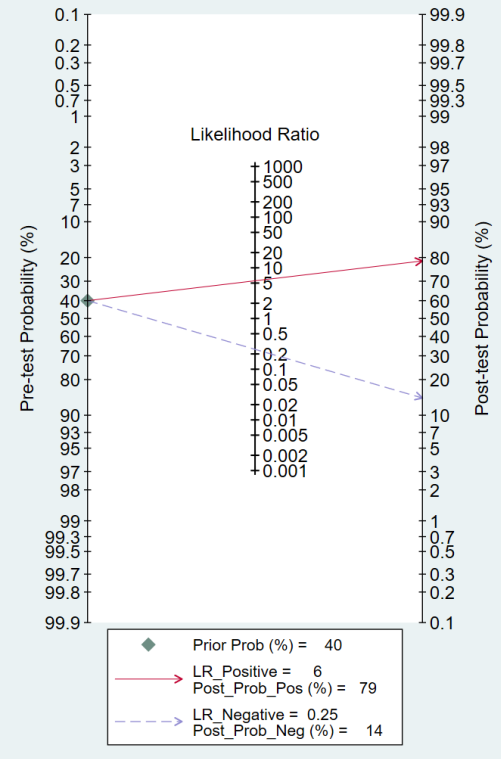


**Figure** **S12** Fagan’s nomogram for DL in detecting ALNM based on conventional US


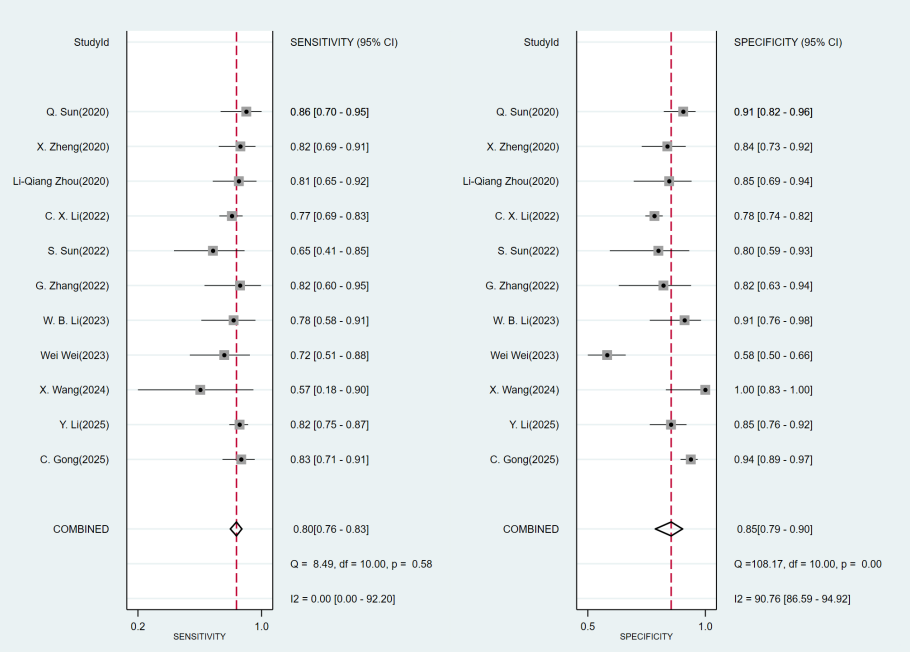


**Figure** **S13** For the internal validation, Forest plot of sensitivity and specificity for DL in detecting ALNM based on conventional US


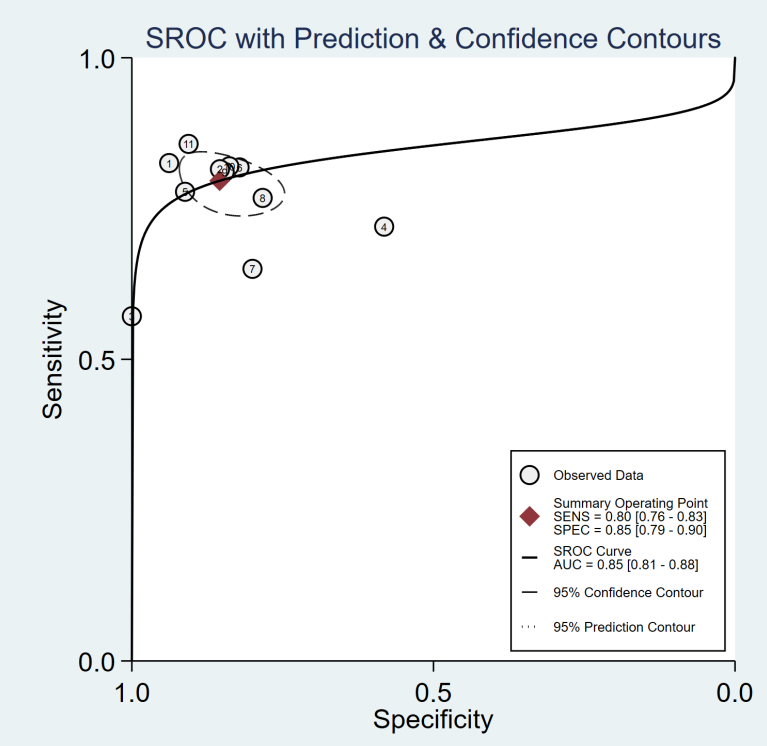


**Figure S14** For the internal validation, SROC curve for DL in detecting ALNM based on conventional US


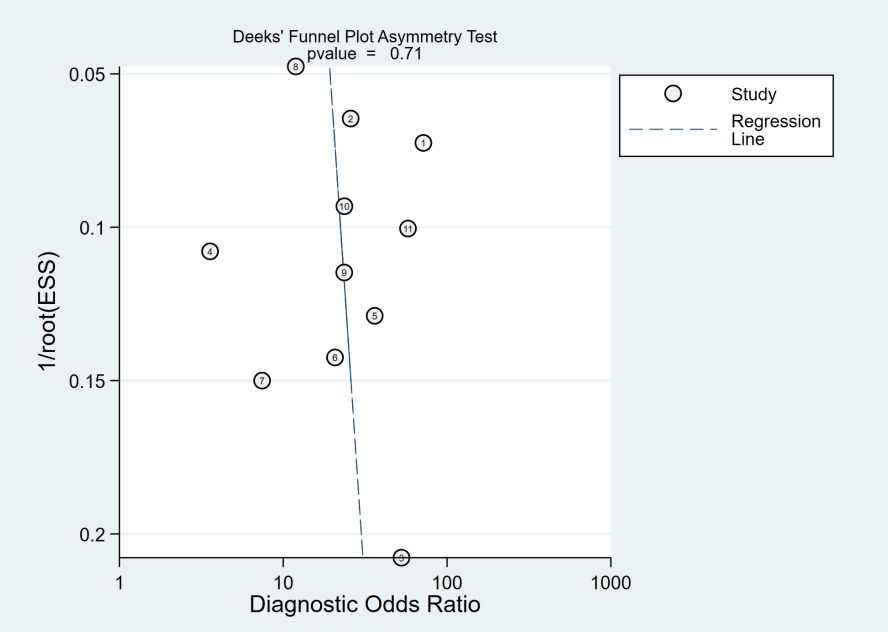


**Figure** **S15** For the internal validation, Deeks' funnel plot for DL in detecting ALNM based on conventional US


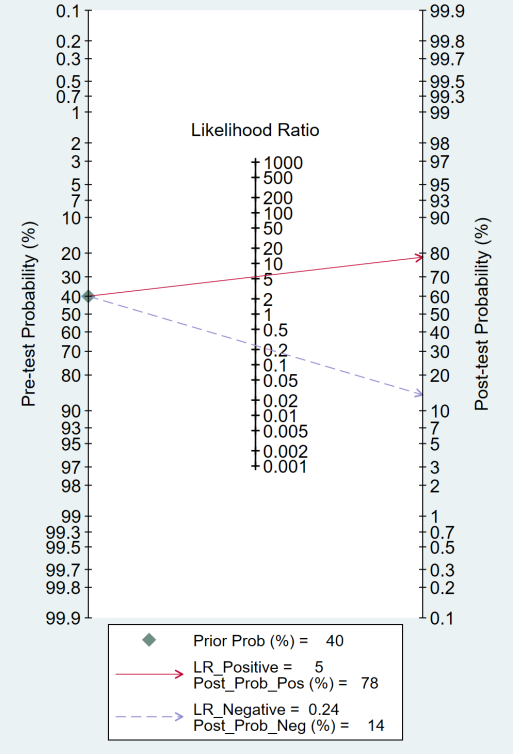


**Figure** **S16** For the internal validation, Fagan’s nomogram for DL in detecting ALNM based on conventional US


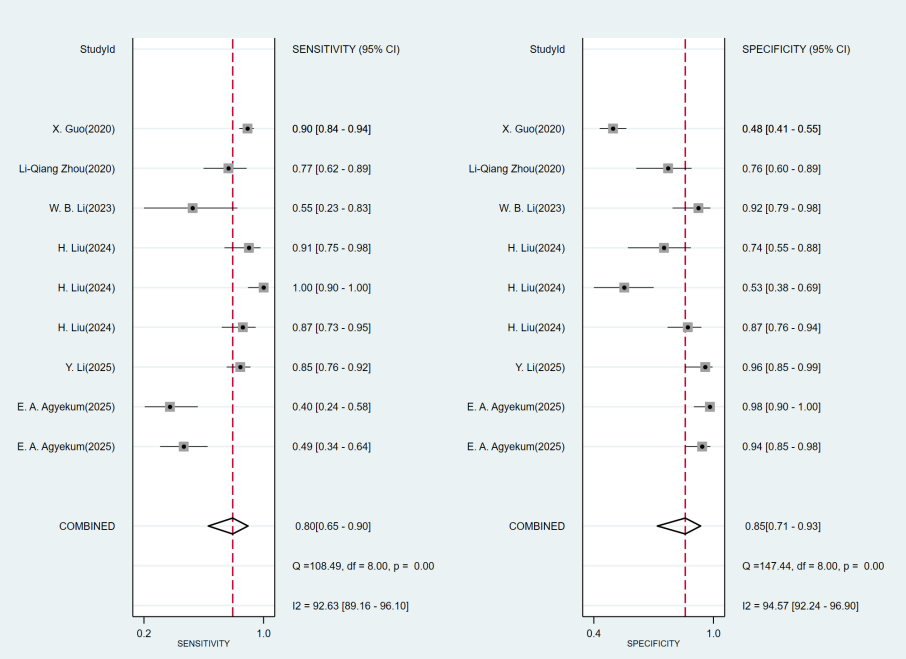


**Figure** **S17** For the external validation, Forest plot of sensitivity and specificity for DL in detecting ALNM based on conventional US


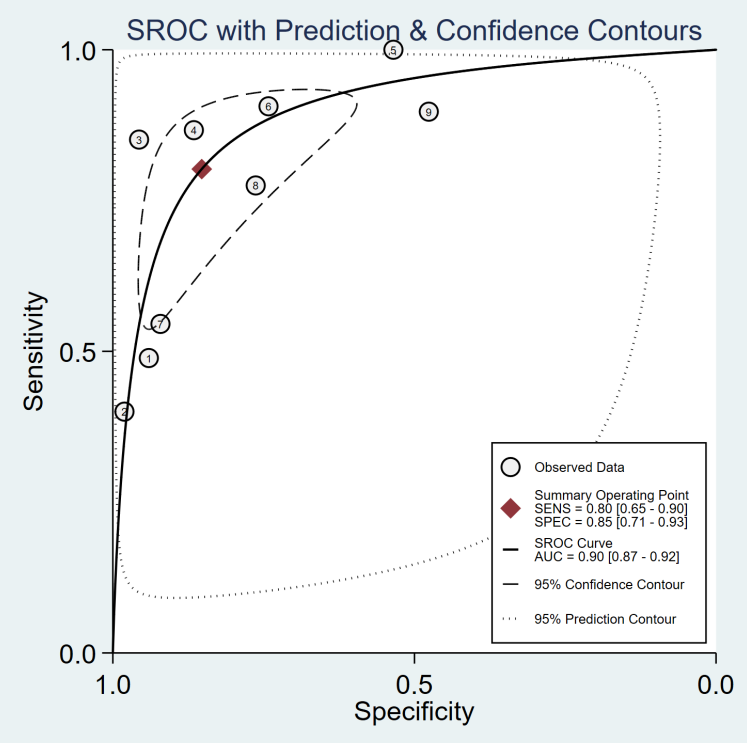


**Figure** **S18** For the external validation, SROC curve for DL in detecting ALNM based on conventional US


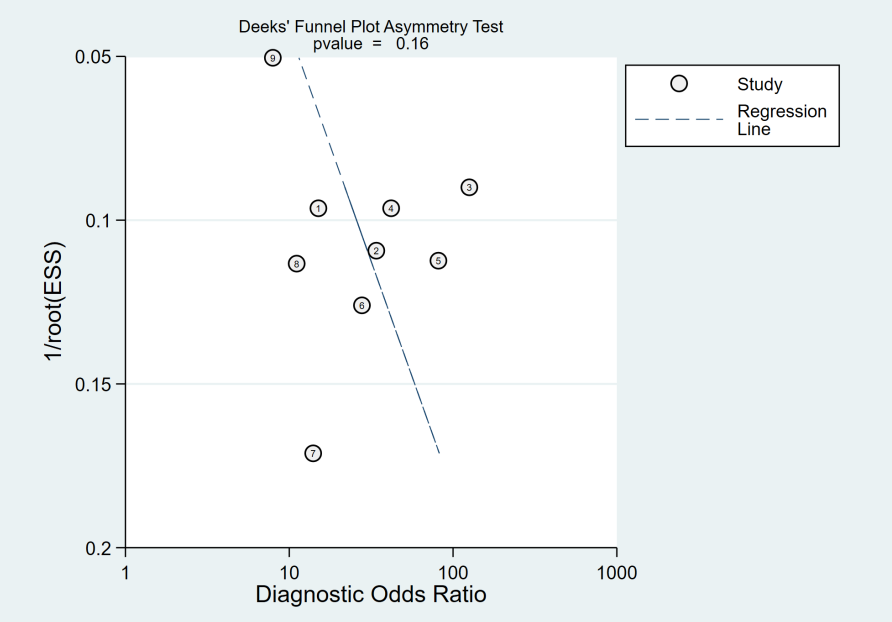


**Figure** **S19** For the external validation, Deeks' funnel plot for DL in detecting ALNM based on conventional US


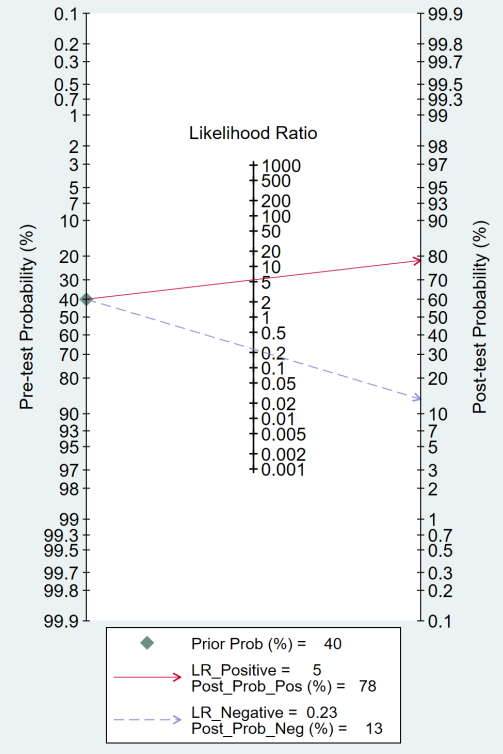


**Figure** **S20** For the external validation, Fagan’s nomogram for DL in detecting ALNM based on conventional US


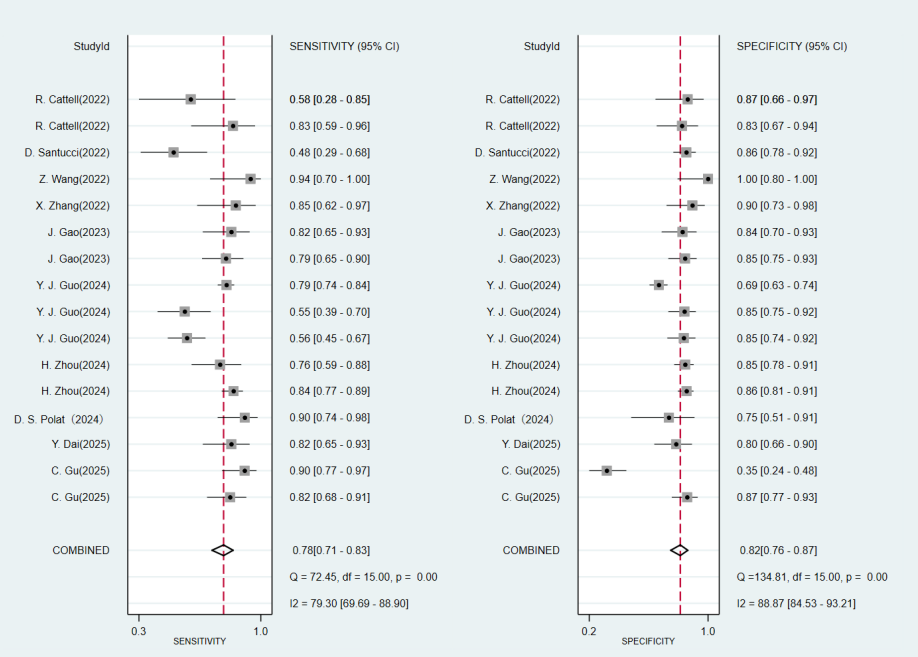


**Figure** **S21** Forest plot of sensitivity and specificity for DL in detecting ALNM based on MRI


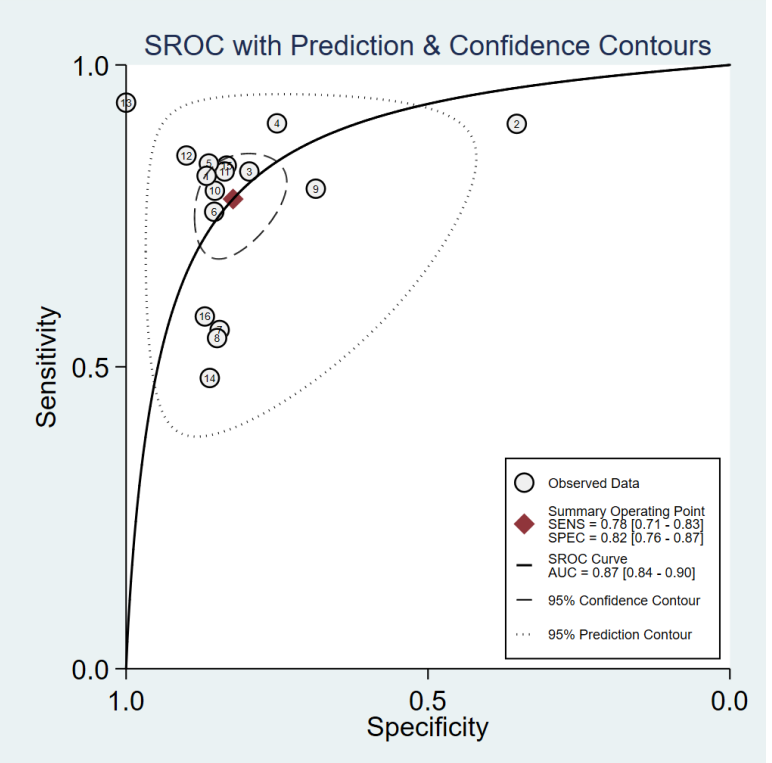


**Figure** **S22** SROC curve for DL in detecting ALNM based on MRI


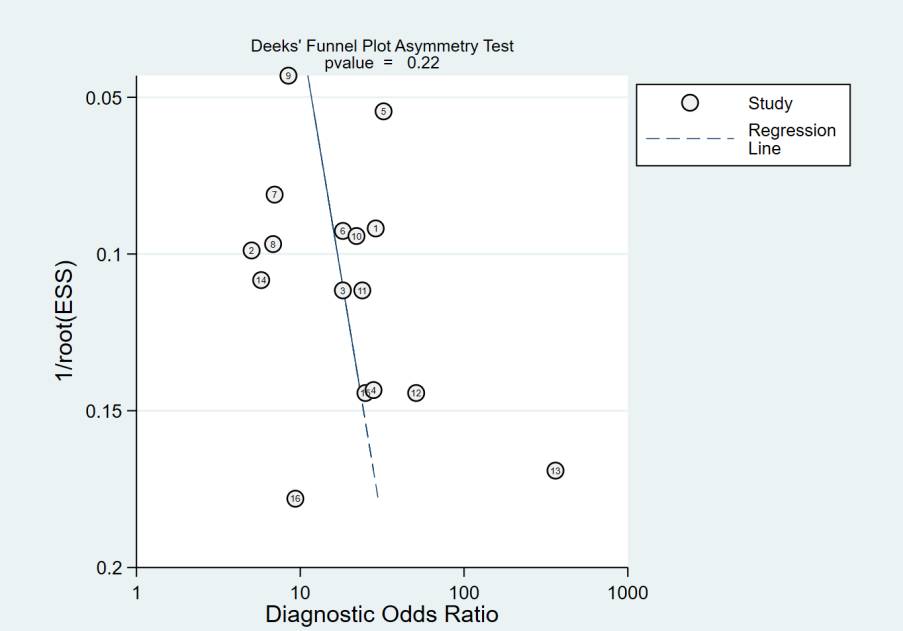


**Figure** **S23** Deeks' funnel plot for DL in detecting ALNM based on MRI


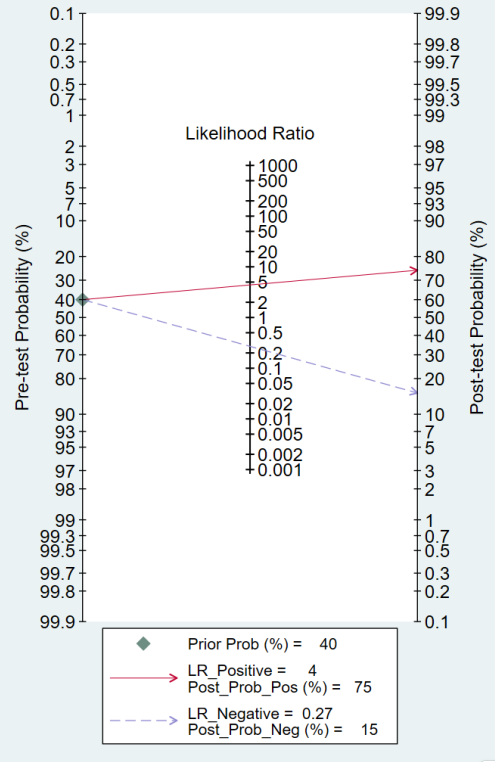


**Figure** **S24** Fagan’s nomogram for DL in detecting ALNM based on MRI


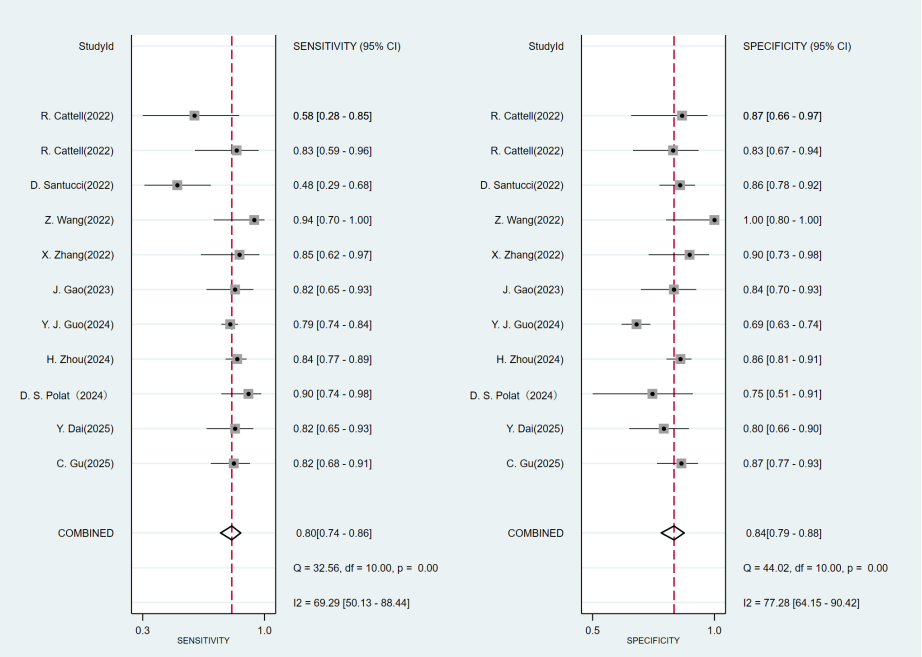


**Figure** **S25** For the internal validation, Forest plot of sensitivity and specificity for DL in detecting ALNM based on MRI


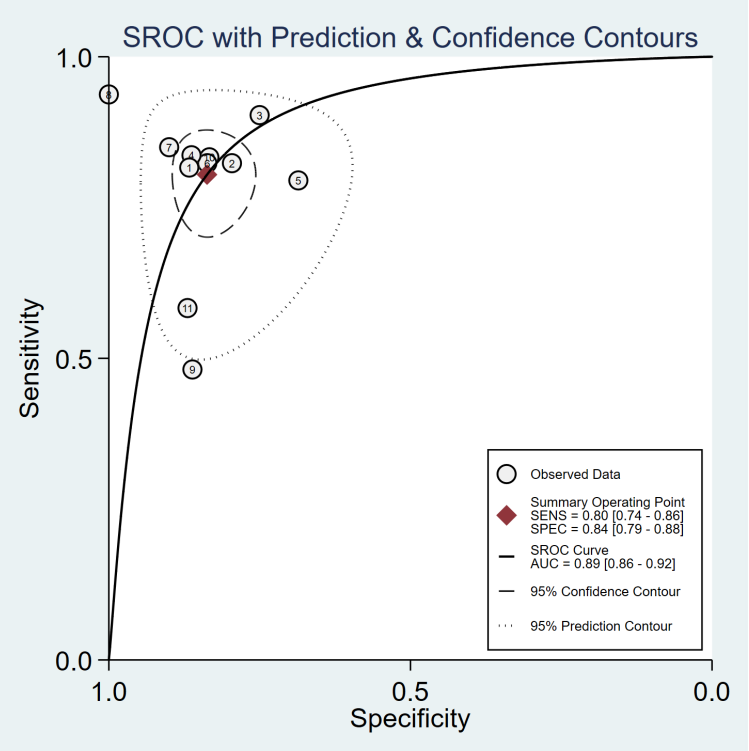


**Figure** **S26** For the internal validation, SROC curve for DL in detecting ALNM based on MRI


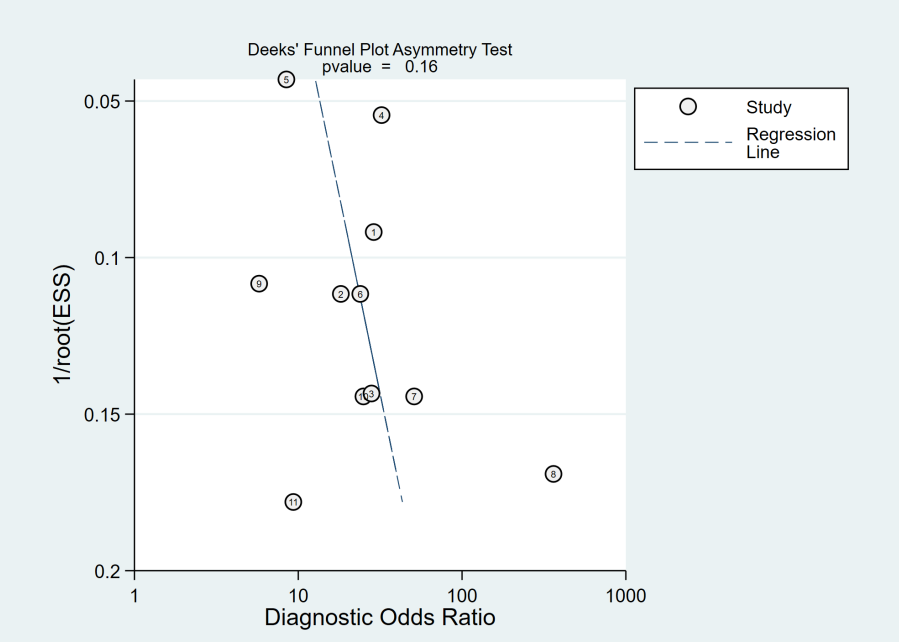


**Figure** **S27** For the internal validation, Deeks' funnel plot for DL in detecting ALNM based on MRI


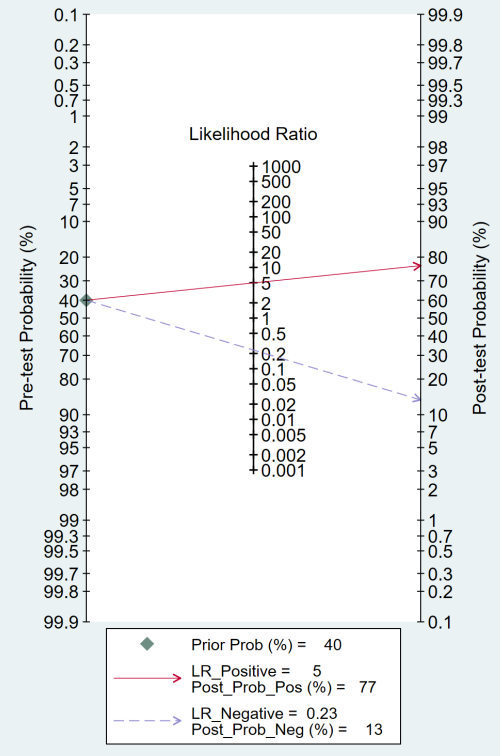


**Figure** **S28** For the internal validation, Fagan’s nomogram for DL in detecting ALNM based on MRI


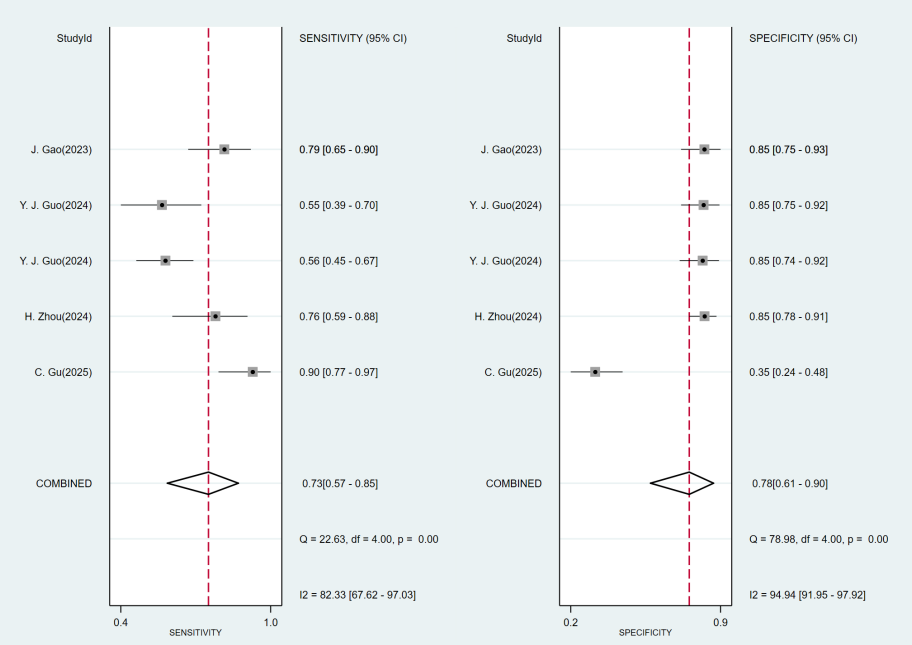


**Figure** **S29** For the external validation, Forest plot of sensitivity and specificity for DL in detecting ALNM based on MRI


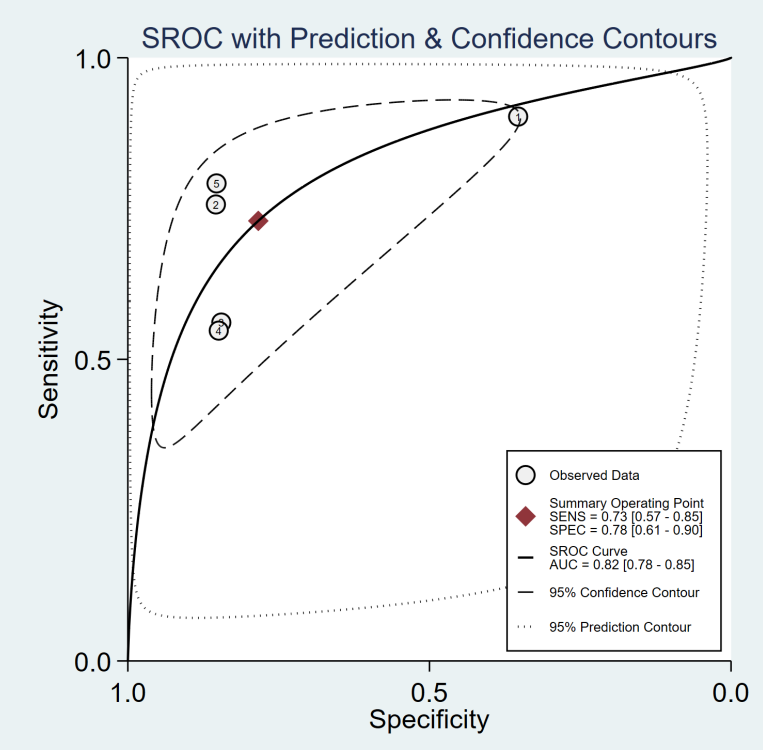


**Figure** **S30** For the external validation, SROC curve for DL in detecting ALNM based on MRI


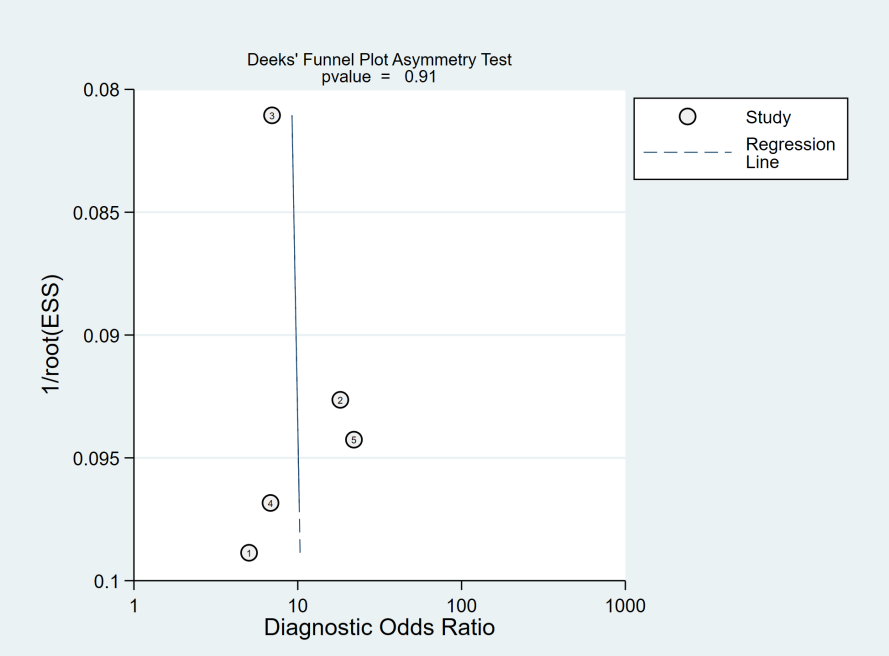


**Figure** **S31** For the external validation, Deeks' funnel plot for DL in detecting ALNM based on MRI


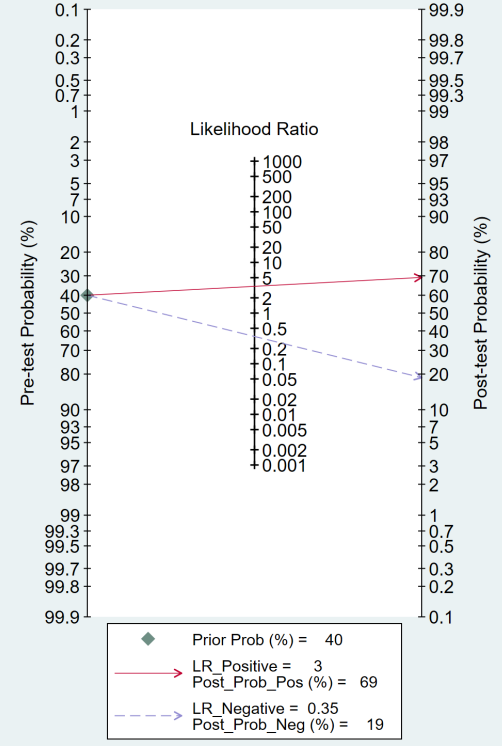


**Figure** **S32** For the external validation, Fagan’s nomogram for DL in detecting ALNM based on MRI

**
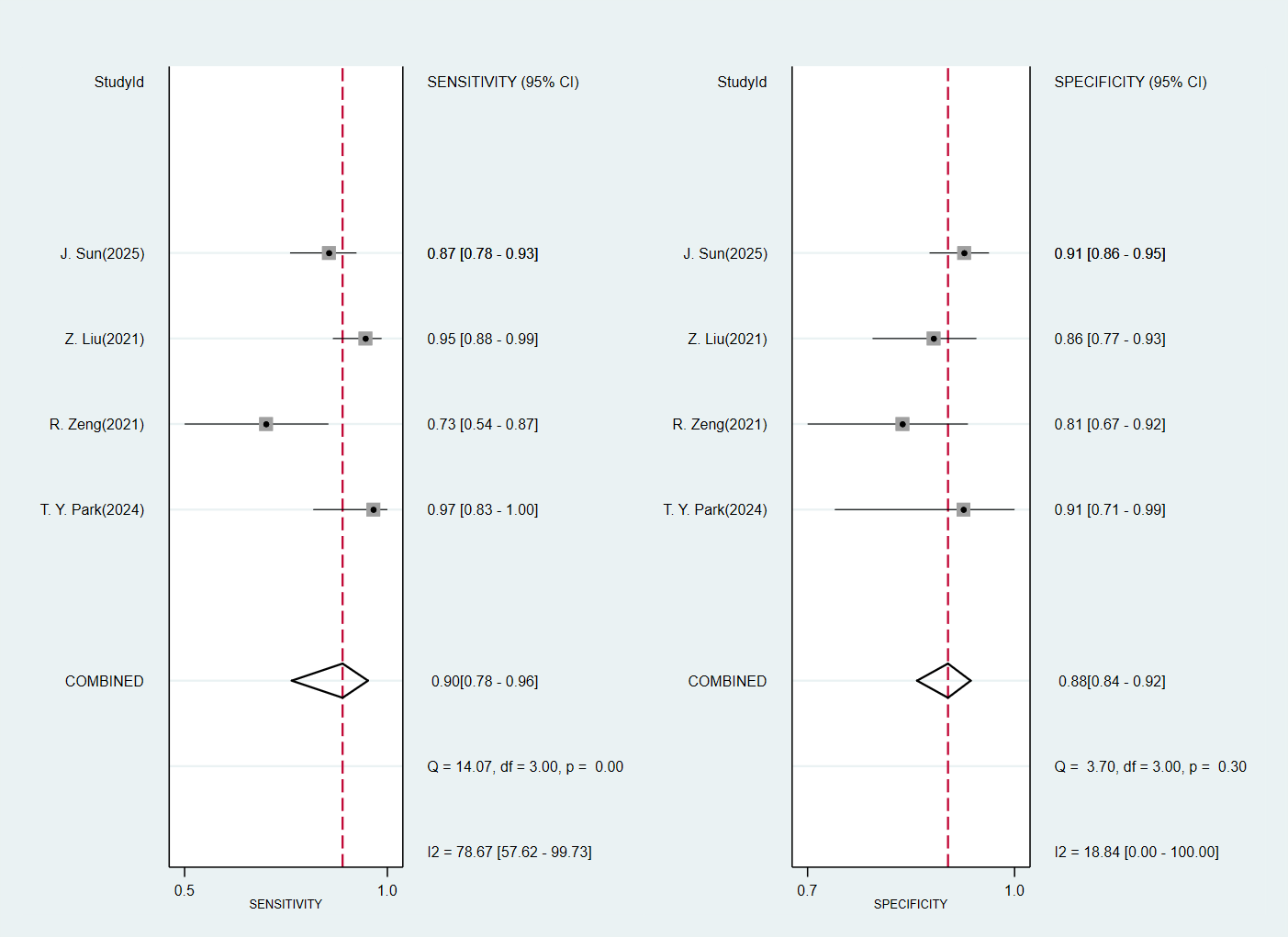
**

**Figure** **S33** Forest plot of sensitivity and specificity for DL in detecting ALNM based on CT

**
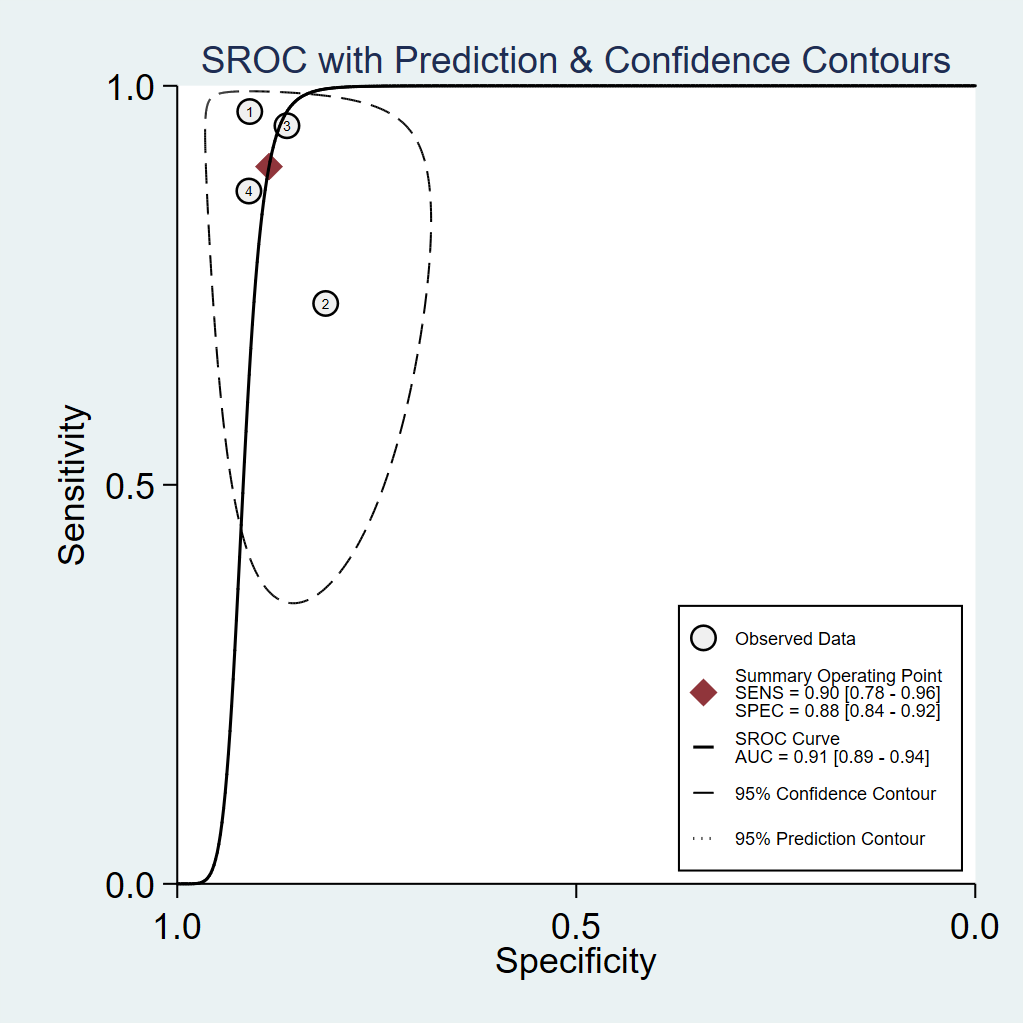
**

**Figure** **S34** SROC curve for DL in detecting ALNM based on CT

**
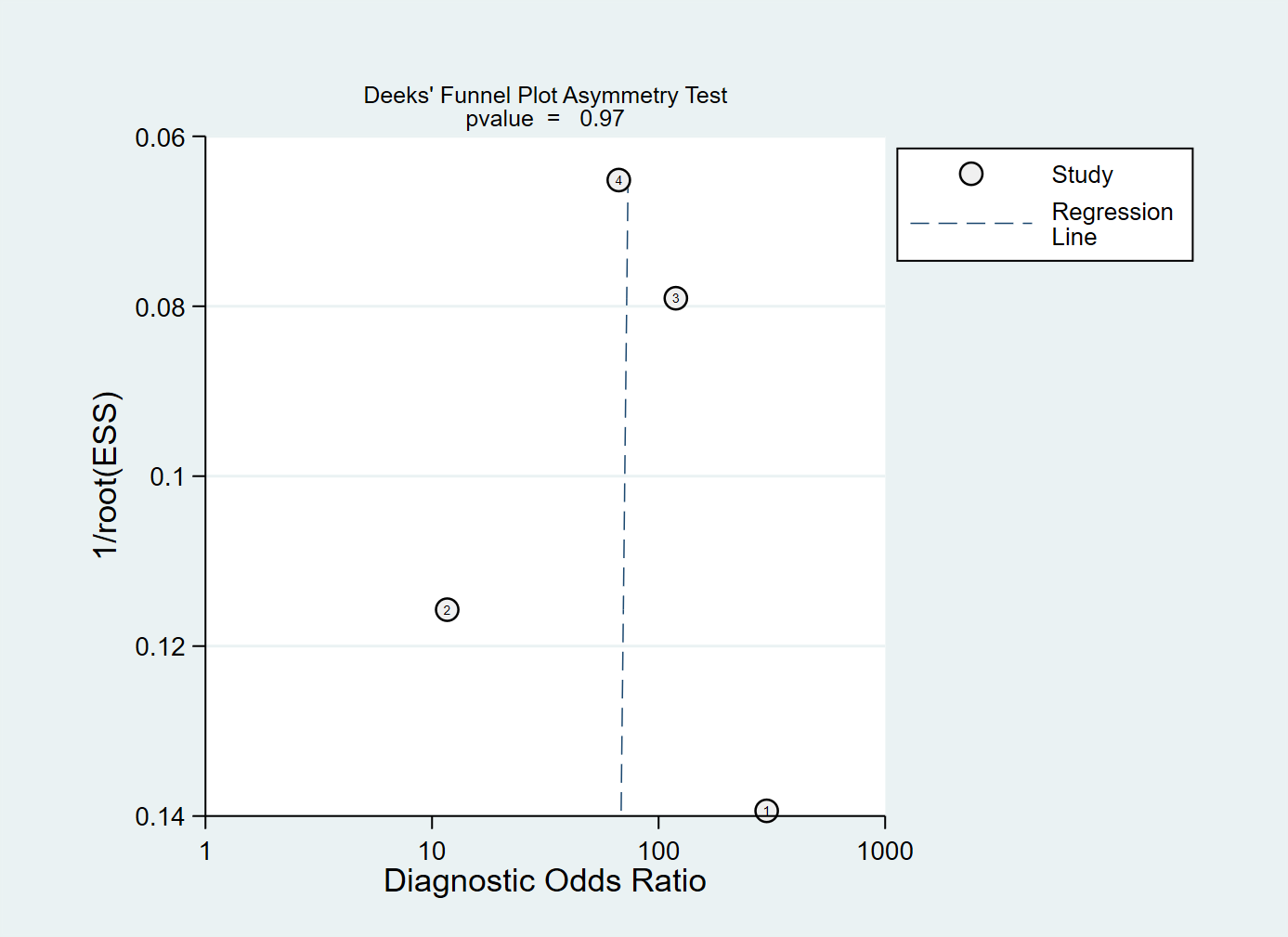
**

**Figure** **S35** Deeks' funnel plot for DL in detecting ALNM based on CT

**
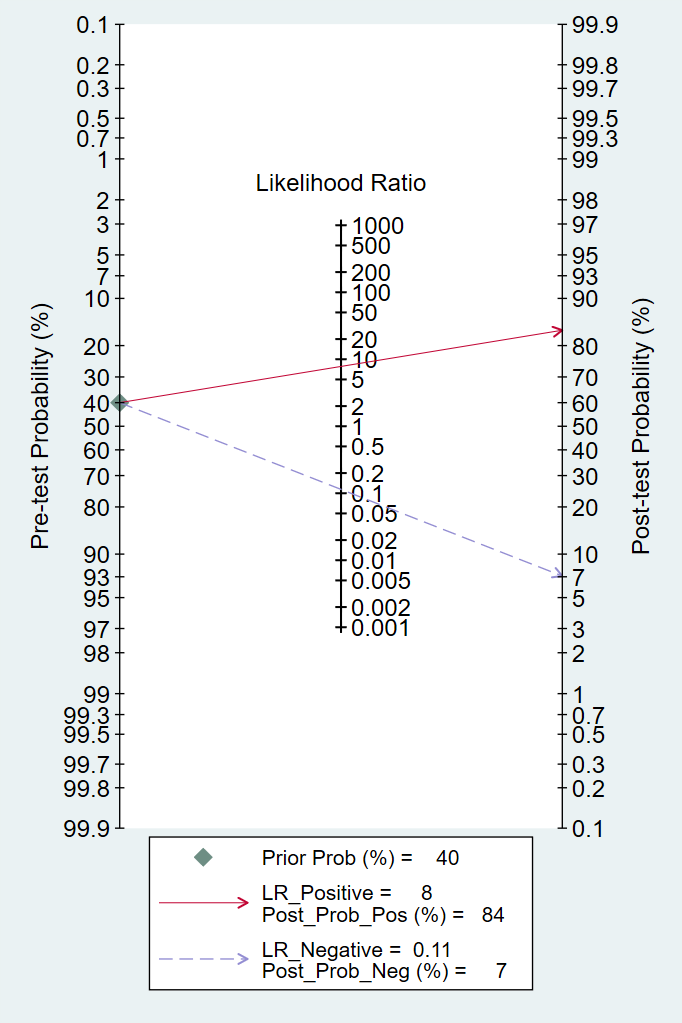
**

**Figure** **S36** Fagan’s nomogram for DL in detecting ALNM based on CT

**
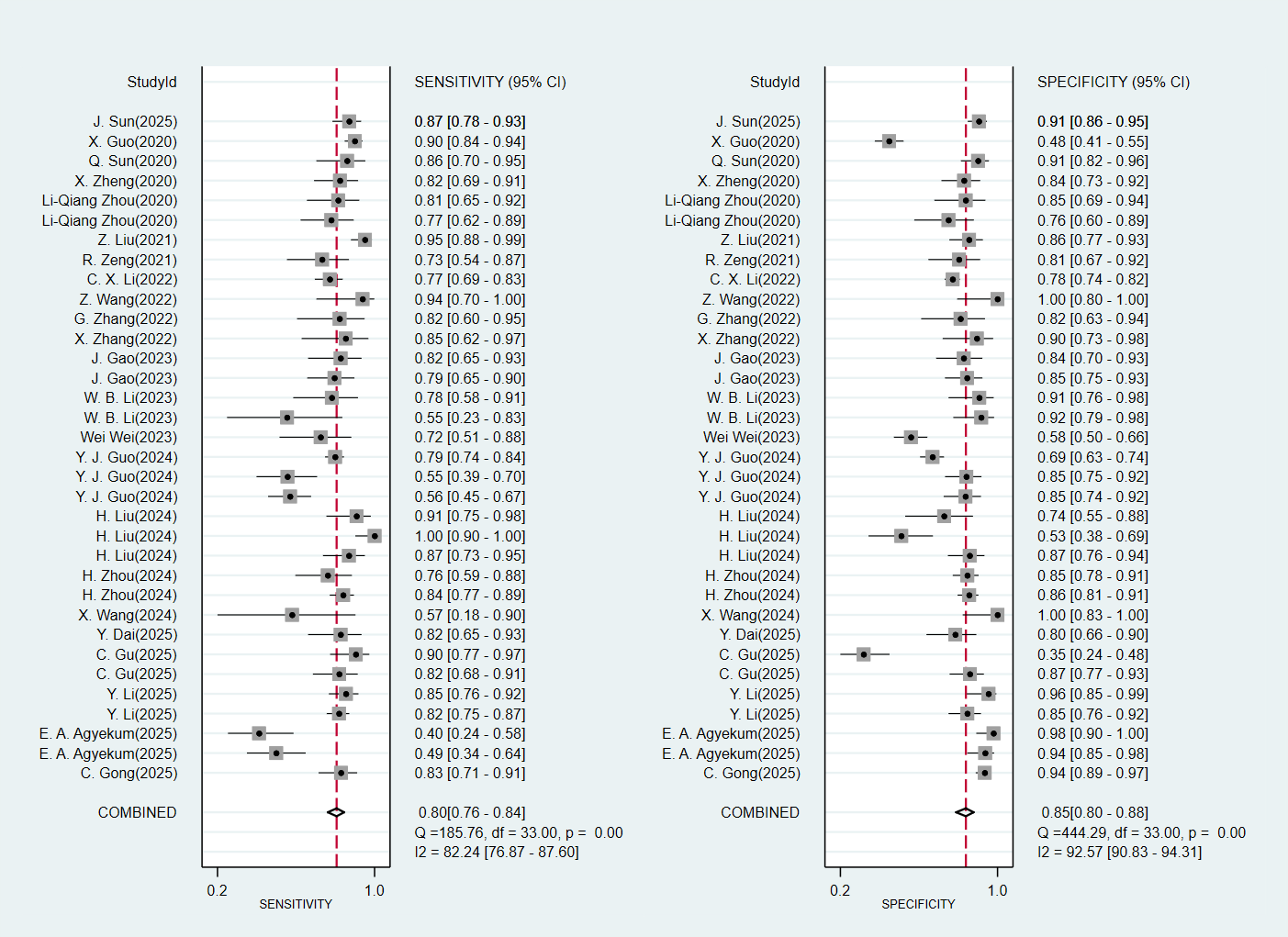
**

**Figure** **S37** Forest plot of sensitivity and specificity for DL in detecting ALNM in Chinese populations

**
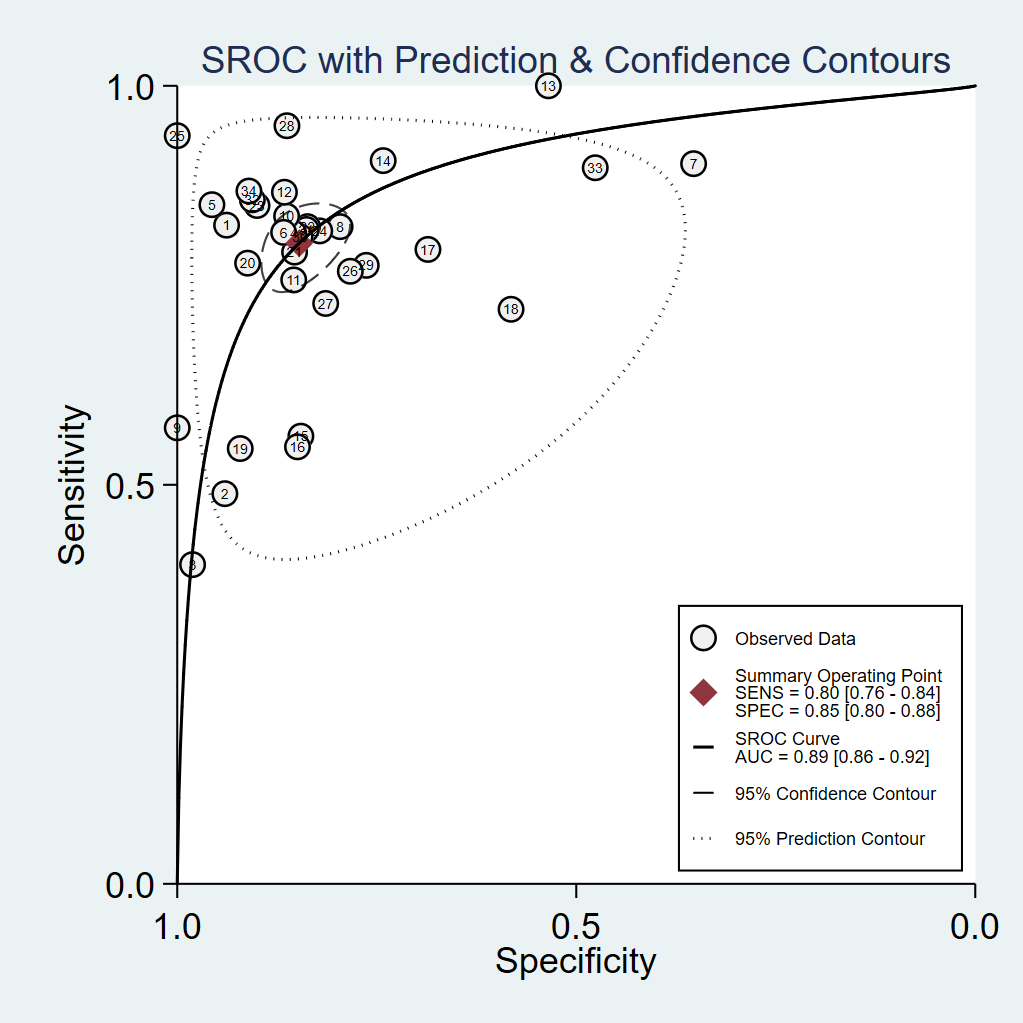
**

**Figure** **S38** SROC curve for DL in detecting ALNM in Chinese populations

**
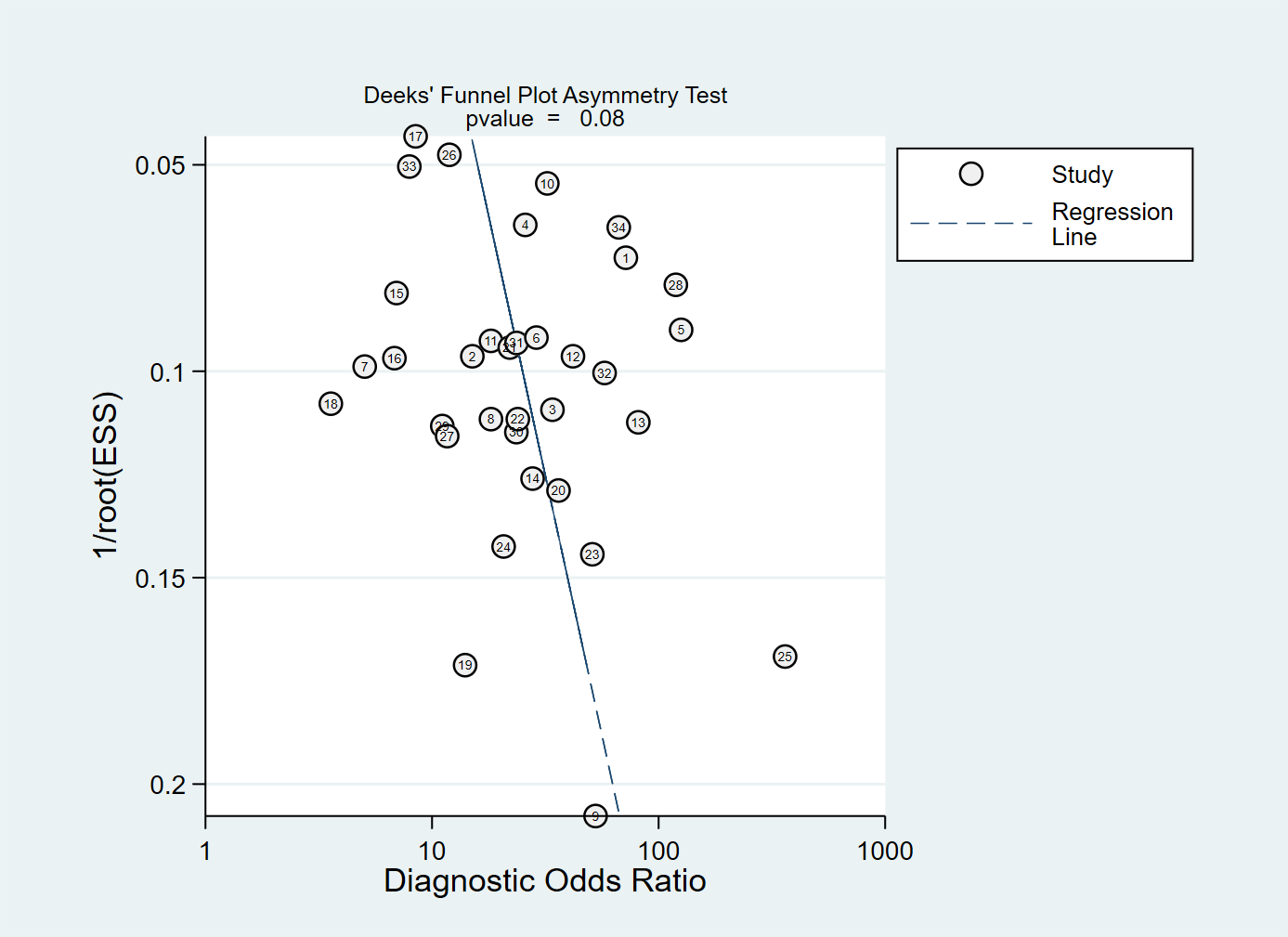
**

**Figure** **S39** Deeks' funnel plot for DL in detecting ALNM in Chinese populations

**
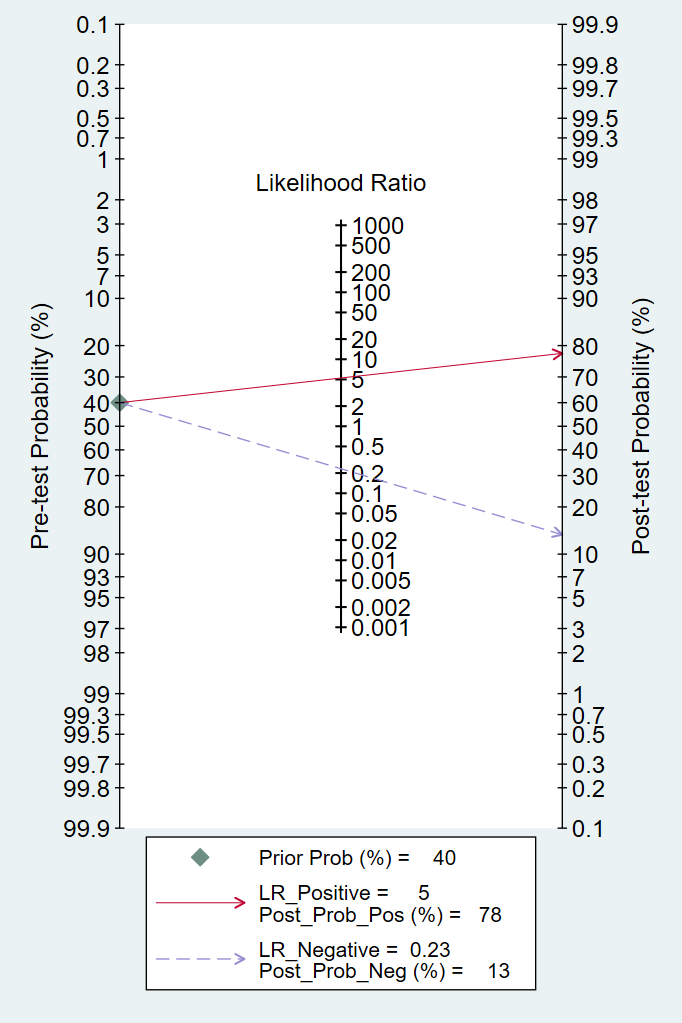
**

**Figure** **S40** Fagan's nomogram for DL in detecting ALNM in Chinese populations


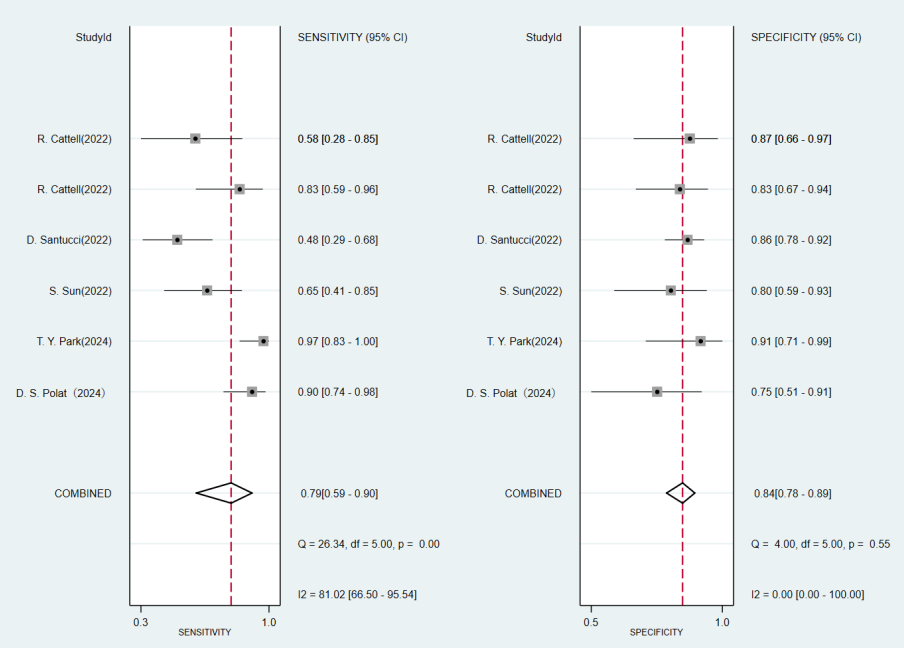


**Figure** **S41** Forest plot of sensitivity and specificity for DL in detecting ALNM in non-Chinese populations


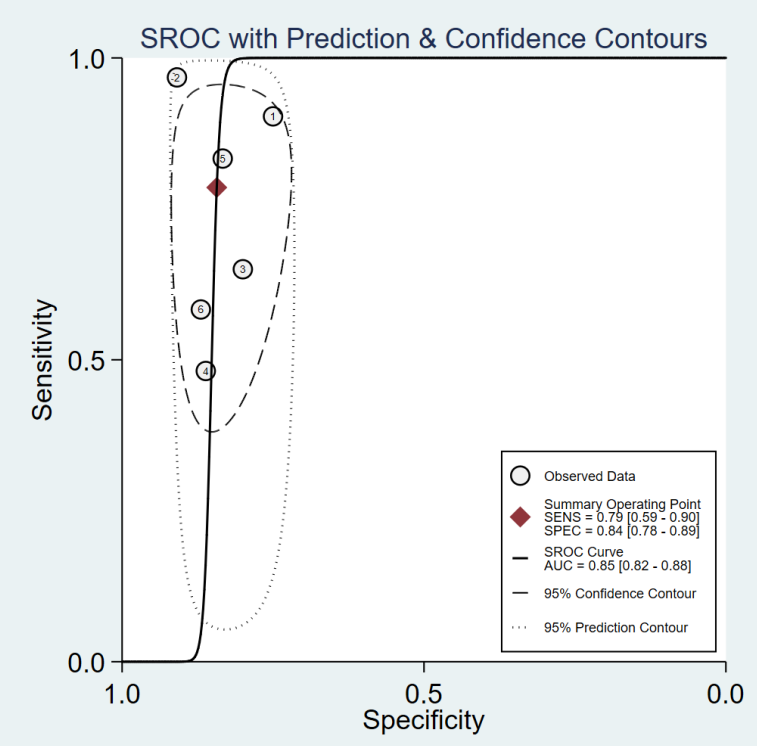


**Figure** **S42** SROC curve for DL in detecting ALNM in non-Chinese populations


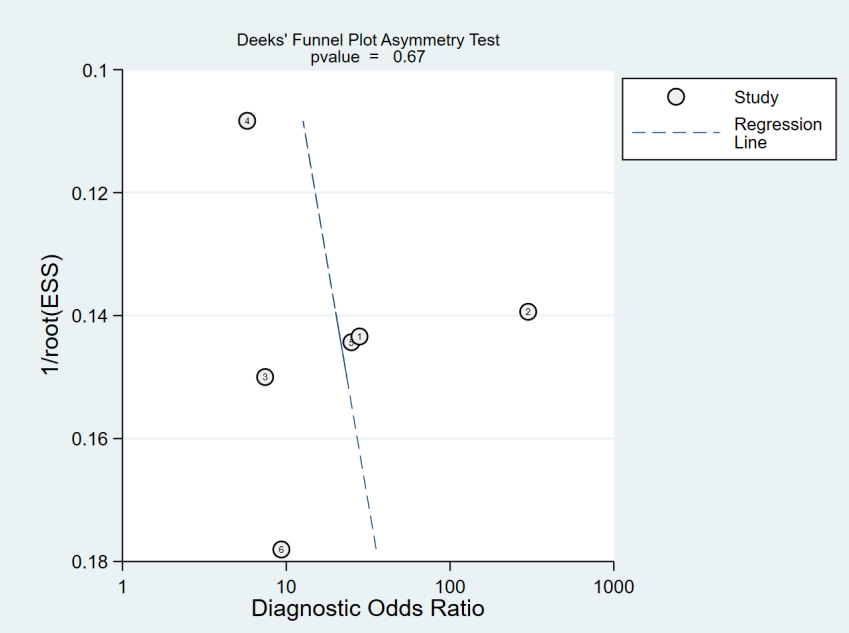


**Figure** **S43** Deeks' funnel plot for DL in detecting ALNM in non-Chinese populations


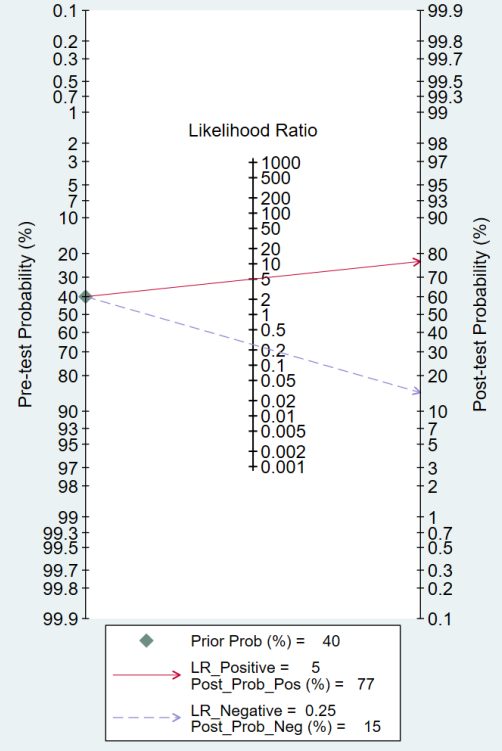


**Figure** **S44** Fagan's nomogram for DL in detecting ALNM in non-Chinese populations


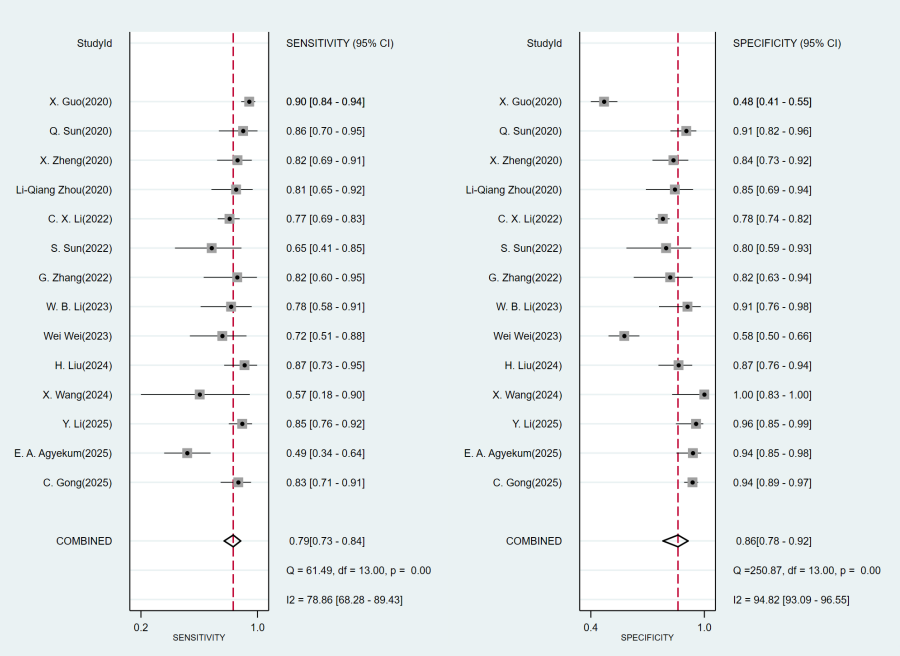


**Figure S45** Forest plot of sensitivity and specificity for DL in detecting ALNM based on conventional US (sensitivity analysis: inclusion of a single diagnostic four-fold table per study)


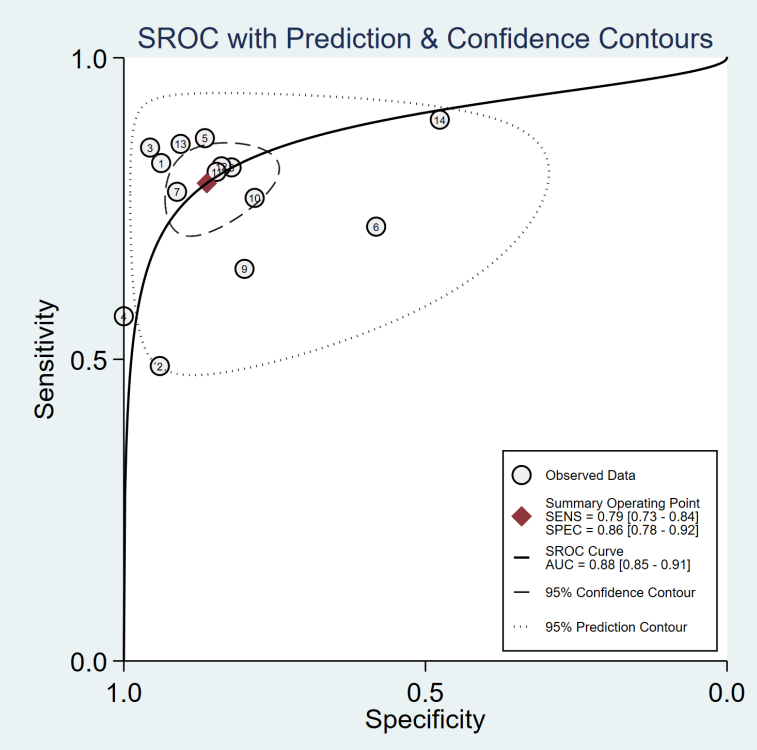


**Figure S46** SROC curve for DL in detecting ALNM based on conventional US (sensitivity analysis: inclusion of a single diagnostic four-fold table per study)


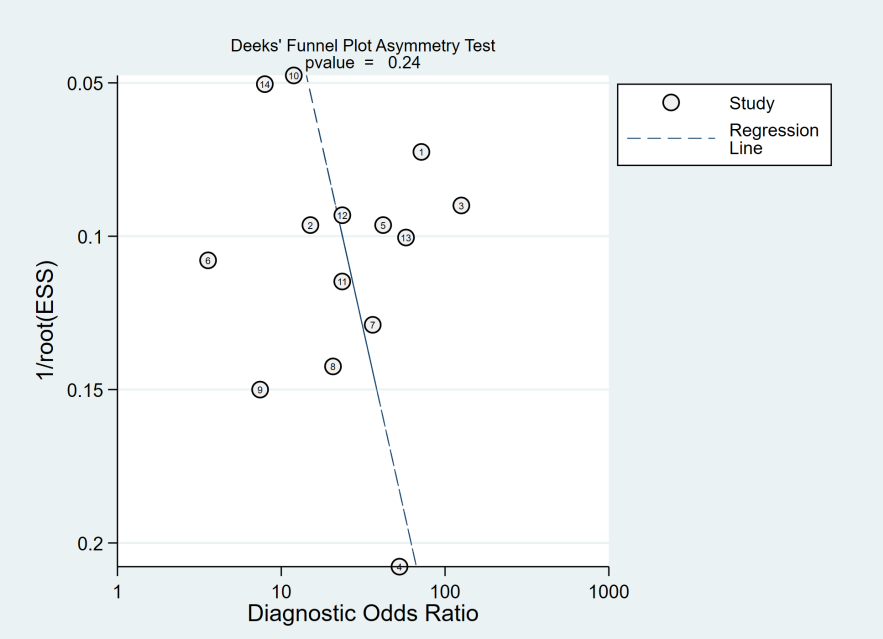


**Figure** **S47** Deeks' funnel plot for DL in detecting ALNM based on conventional US (sensitivity analysis: inclusion of a single diagnostic four-fold table per study)


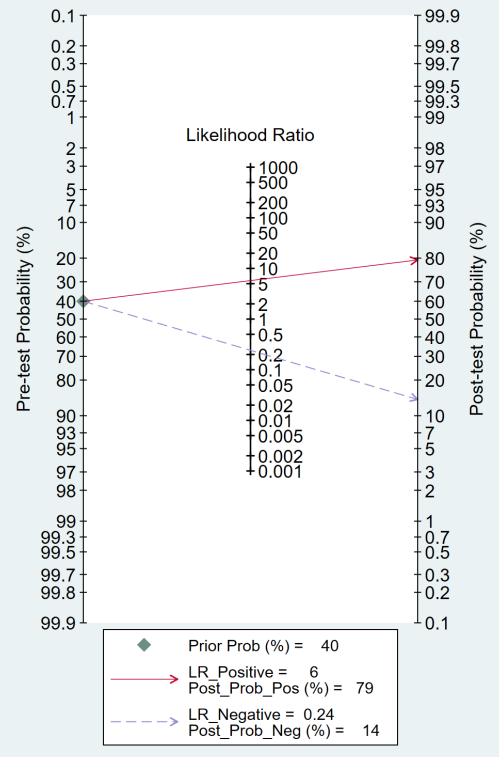


**Figure** **S48** Fagan’s nomogram for DL in detecting ALNM based on conventional US (sensitivity analysis: inclusion of a single diagnostic four-fold table per study)


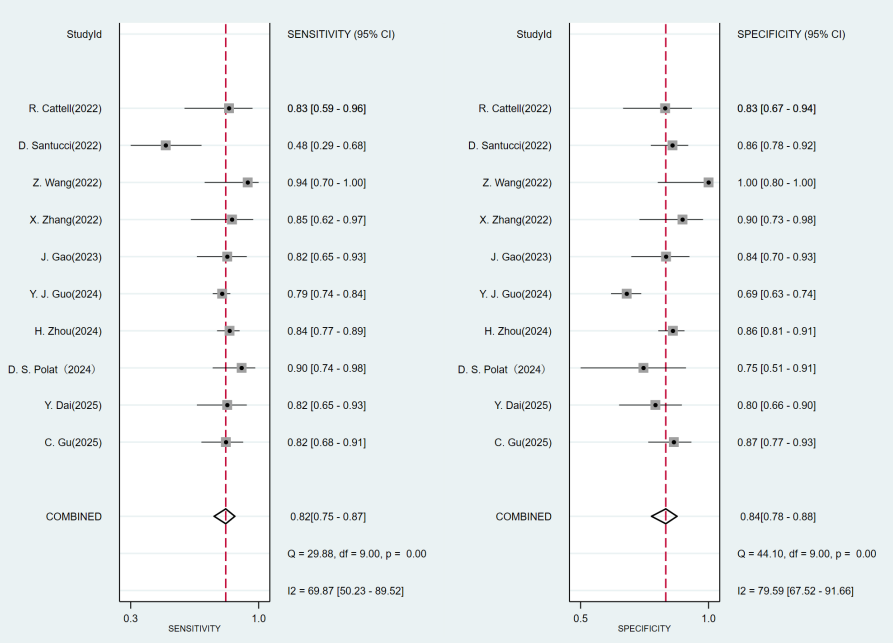


**Figure S49** Forest plot of sensitivity and specificity for DL in detecting ALNM based on conventional MRI (sensitivity analysis: inclusion of a single diagnostic four-fold table per study)


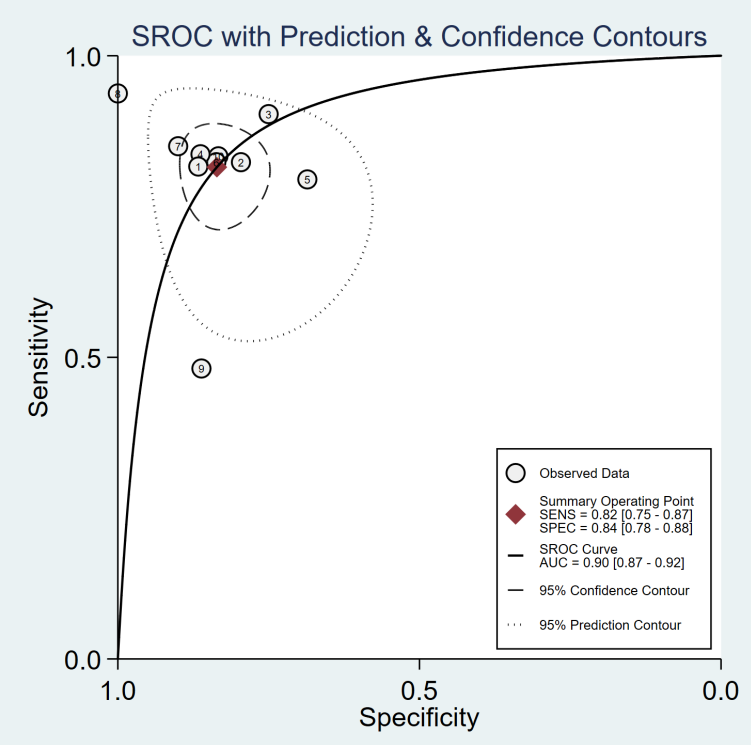


**Figure S50** SROC curve for DL in detecting ALNM based on conventional MRI (sensitivity analysis: inclusion of a single diagnostic four-fold table per study)


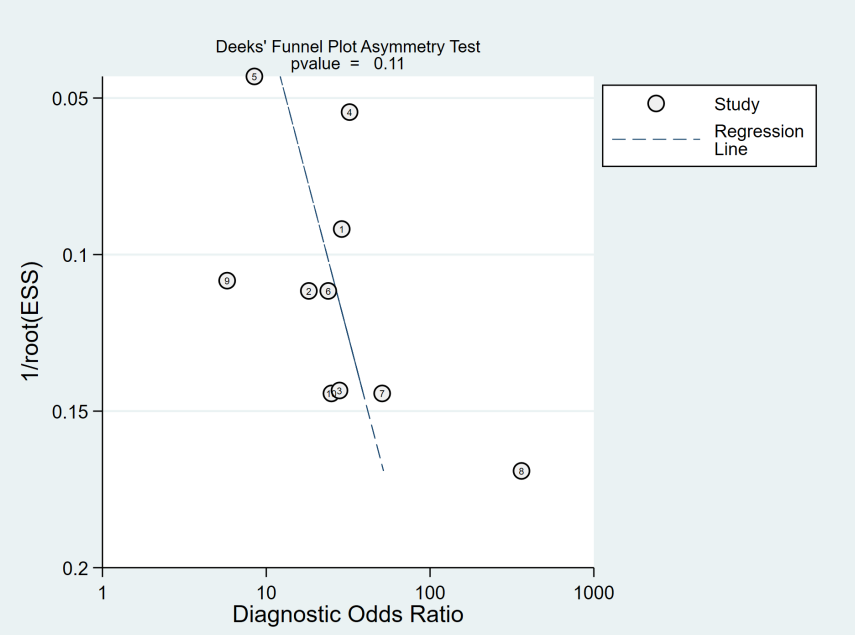


**Figure** **S51** Deeks' funnel plot for DL in detecting ALNM based on conventional MRI (sensitivity analysis: inclusion of a single diagnostic four-fold table per study)


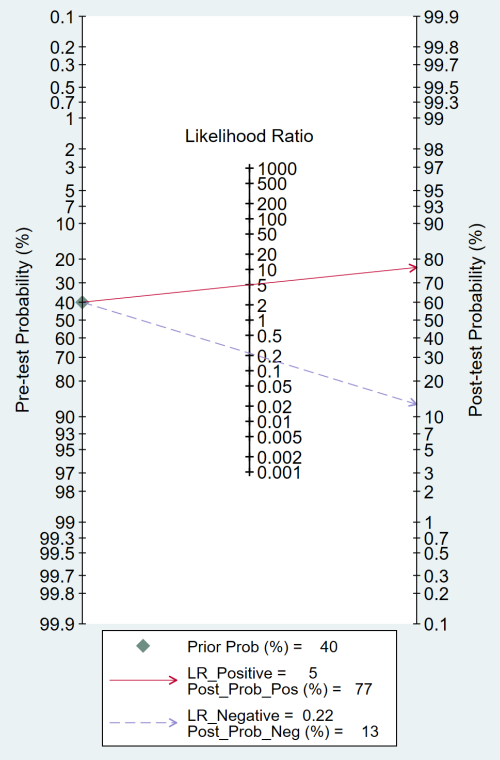


**Figure** **S52** Fagan’s nomogram for DL in detecting ALNM based on conventional

MRI (sensitivity analysis: inclusion of a single diagnostic four-fold table per study)


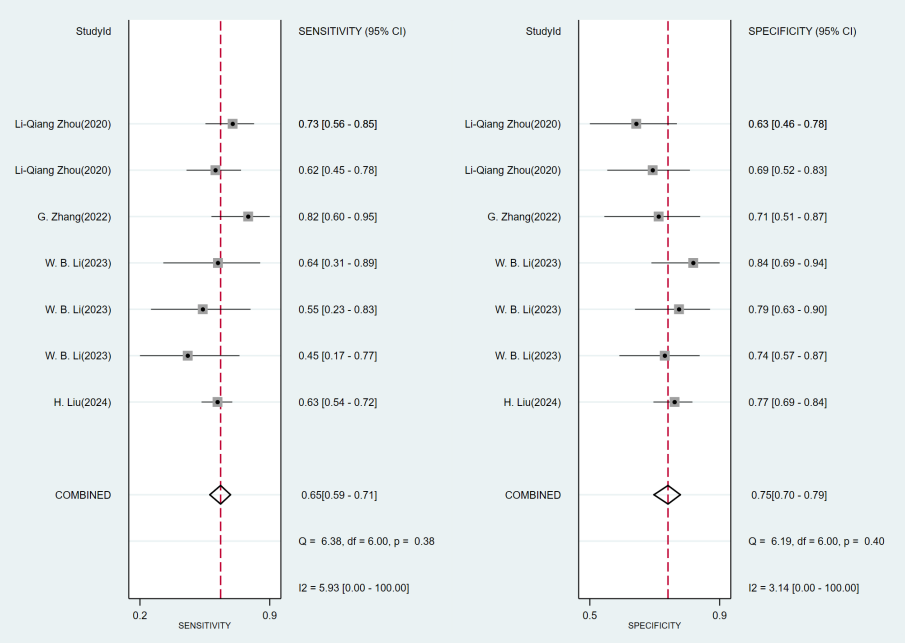


**Figure** **S53** Forest plot of sensitivity and specificity for clinical experts in detecting ALNM based on US


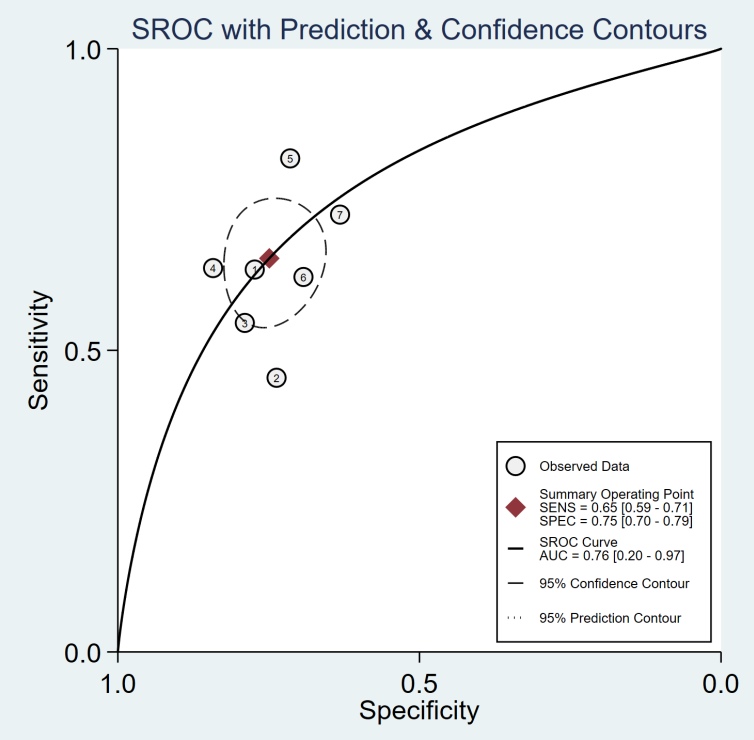


**Figure** **S54** SROC curve for clinical experts in detecting ALNM based on US


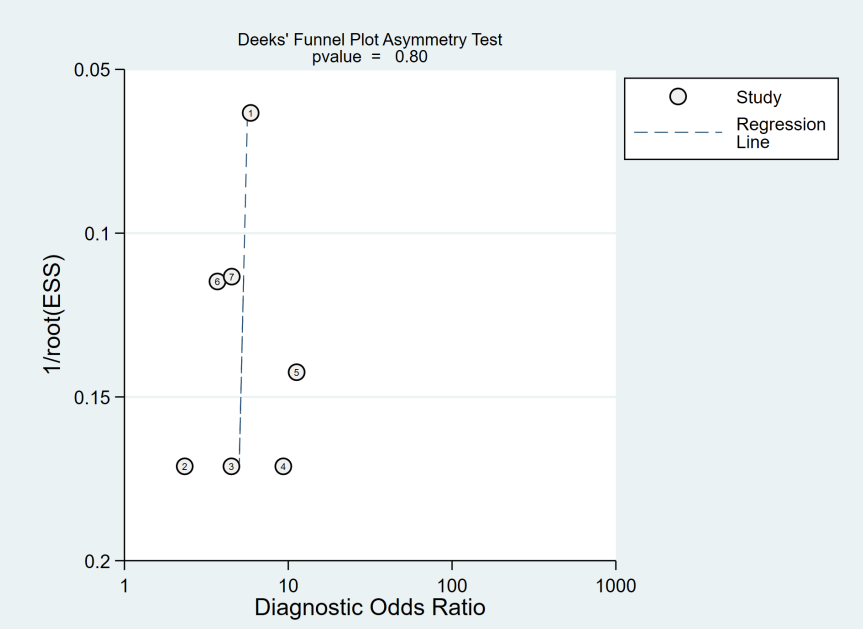


**Figure** **S55** Deeks' funnel plot for clinical experts in detecting ALNM based on US


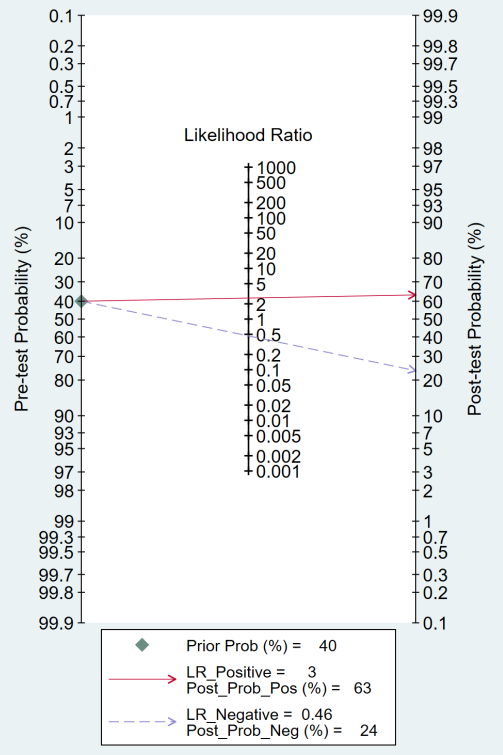


**Figure** **S56** Fagan's nomogram for clinical experts in detecting ALNM based on US

**Table S1** Detailed electronic search strategy. This table shows the complete search syntax, including all keywords and controlled vocabulary terms. It was executed in the PubMed database and adapted for other databases, including Web of Science, Embase, and the Cochrane Library, up to May 26, 2024.

**1.Pubmed**

| Search number | Query | Results |
| --- | --- | --- |
| #1 | "Breast Neoplasms"[Mesh] | 353785 |
| #2 | (((((((((((((((((((((((((((((((((((((((((Breast Neoplasms[Title/Abstract]) OR (Breast Neoplasm[Title/Abstract])) OR (Breast Tumors[Title/Abstract])) OR (Breast Tumor[Title/Abstract])) OR (Breast Cancer[Title/Abstract])) OR (Mammary Cancer[Title/Abstract])) OR (Mammary Cancers[Title/Abstract])) OR (Malignant Neoplasm of Breast[Title/Abstract])) OR (Breast Malignant Neoplasm[Title/Abstract])) OR (Breast Malignant Neoplasms[Title/Abstract])) OR (Malignant Tumor of Breast[Title/Abstract])) OR (Breast Malignant Tumor[Title/Abstract])) OR (Breast Malignant Tumors[Title/Abstract])) OR (Cancer of Breast[Title/Abstract])) OR (Cancer of the Breast[Title/Abstract])) OR (Human Mammary Carcinomas[Title/Abstract])) OR (Human Mammary Carcinoma[Title/Abstract])) OR (Breast Carcinoma[Title/Abstract])) OR (Breast Carcinomas[Title/Abstract])) OR (breast tumor[Title/Abstract])) OR (breast gland tumor[Title/Abstract])) OR (breast gland tumour[Title/Abstract])) OR (breast mass[Title/Abstract])) OR (mamma tumor[Title/Abstract])) OR (mamma tumour[Title/Abstract])) OR (mammary gland neoplasia[Title/Abstract])) OR (mammary gland neoplasm[Title/Abstract])) OR (mammary gland tumor[Title/Abstract])) OR (mammary gland tumorigenesis[Title/Abstract])) OR (mammary neoplasia[Title/Abstract])) OR (mammary neoplasm[Title/Abstract])) OR (mammary neoplasms[Title/Abstract])) OR (mammary tumorigenesis[Title/Abstract])) OR (neoplasia of the breast[Title/Abstract])) OR (neoplasm of the breast[Title/Abstract])) OR (neoplasm of the mammary gland[Title/Abstract])) OR (neoplastic breast[Title/Abstract])) OR (neoplastic mammary[Title/Abstract])) OR (tumor of the breast[Title/Abstract])) OR (tumor of the mammary gland[Title/Abstract])) OR (tumorigenesis of the breast[Title/Abstract])) OR (tumorigenesis of the mammary gland[Title/Abstract]) | 399876 |
| #3 | ("Breast Neoplasms"[Mesh]) OR ((((((((((((((((((((((((((((((((((((((((((Breast Neoplasms[Title/Abstract]) OR (Breast Neoplasm[Title/Abstract])) OR (Breast Tumors[Title/Abstract])) OR (Breast Tumor[Title/Abstract])) OR (Breast Cancer[Title/Abstract])) OR (Mammary Cancer[Title/Abstract])) OR (Mammary Cancers[Title/Abstract])) OR (Malignant Neoplasm of Breast[Title/Abstract])) OR (Breast Malignant Neoplasm[Title/Abstract])) OR (Breast Malignant Neoplasms[Title/Abstract])) OR (Malignant Tumor of Breast[Title/Abstract])) OR (Breast Malignant Tumor[Title/Abstract])) OR (Breast Malignant Tumors[Title/Abstract])) OR (Cancer of Breast[Title/Abstract])) OR (Cancer of the Breast[Title/Abstract])) OR (Human Mammary Carcinomas[Title/Abstract])) OR (Human Mammary Carcinoma[Title/Abstract])) OR (Breast Carcinoma[Title/Abstract])) OR (Breast Carcinomas[Title/Abstract])) OR (breast tumor[Title/Abstract])) OR (breast gland tumor[Title/Abstract])) OR (breast gland tumour[Title/Abstract])) OR (breast mass[Title/Abstract])) OR (mamma tumor[Title/Abstract])) OR (mamma tumour[Title/Abstract])) OR (mammary gland neoplasia[Title/Abstract])) OR (mammary gland neoplasm[Title/Abstract])) OR (mammary gland tumor[Title/Abstract])) OR (mammary gland tumorigenesis[Title/Abstract])) OR (mammary neoplasia[Title/Abstract])) OR (mammary neoplasm[Title/Abstract])) OR (mammary neoplasms[Title/Abstract])) OR (mammary tumorigenesis[Title/Abstract])) OR (neoplasia of the breast[Title/Abstract])) OR (neoplasm of the breast[Title/Abstract])) OR (neoplasm of the mammary gland[Title/Abstract])) OR (neoplastic breast[Title/Abstract])) OR (neoplastic mammary[Title/Abstract])) OR (tumor of the breast[Title/Abstract])) OR (tumor of the mammary gland[Title/Abstract])) OR (tumorigenesis of the breast[Title/Abstract])) OR (tumorigenesis of the mammary gland[Title/Abstract])) | 477701 |
| #4 | machine learning[MeSH Terms] | 68745 |
| #5 | (((((((((((((((((machine learning[Title/Abstract]) OR (Transfer Learning[Title/Abstract])) OR (Deep learning[Title/Abstract])) OR (Ensemble Learning[Title/Abstract])) OR (artificial intelligence[Title/Abstract])) OR (neural network[Title/Abstract])) OR (neural networks[Title/Abstract])) OR (K-Nearest Neighbor[Title/Abstract])) OR (CNN[Title/Abstract]))) OR (AlexNet[Title/Abstract])) OR (VGGNet[Title/Abstract])) OR (ResNet[Title/Abstract])) OR (GoogLeNet[Title/Abstract])) OR (Radiomics[Title/Abstract])) OR (Radiomic[Title/Abstract])) OR (radiogenomic[Title/Abstract])) OR (Texture[Title/Abstract]) | 313258 |
| #6 | (machine learning[MeSH Terms]) OR ((((((((((((((((((machine learning[Title/Abstract]) OR (Transfer Learning[Title/Abstract])) OR (Deep learning[Title/Abstract])) OR (Ensemble Learning[Title/Abstract])) OR (artificial intelligence[Title/Abstract])) OR (neural network[Title/Abstract])) OR (neural networks[Title/Abstract])) OR (K-Nearest Neighbor[Title/Abstract])) OR (CNN[Title/Abstract]))) OR (AlexNet[Title/Abstract])) OR (VGGNet[Title/Abstract])) OR (ResNet[Title/Abstract])) OR (GoogLeNet[Title/Abstract])) OR (Radiomics[Title/Abstract])) OR (Radiomic[Title/Abstract])) OR (radiogenomic[Title/Abstract])) OR (Texture[Title/Abstract])) | 322933 |
| #7 | Lymphatic Metastasis[MeSH Terms] | 98957 |
| #8 | (((Lymphatic Metastasis[Title/Abstract]) OR (Lymphatic Metastases[Title/Abstract])) OR (Lymph Node Metastasis[Title/Abstract])) OR (Lymph Node Metastases[Title/Abstract]) | 67677 |
| #9 | (Lymphatic Metastasis[MeSH Terms]) OR ((((Lymphatic Metastasis[Title/Abstract]) OR (Lymphatic Metastases[Title/Abstract])) OR (Lymph Node Metastasis[Title/Abstract])) OR (Lymph Node Metastases[Title/Abstract])) | 133635 |
| #10 | ((("Breast Neoplasms"[Mesh]) OR ((((((((((((((((((((((((((((((((((((((((((Breast Neoplasms[Title/Abstract]) OR (Breast Neoplasm[Title/Abstract])) OR (Breast Tumors[Title/Abstract])) OR (Breast Tumor[Title/Abstract])) OR (Breast Cancer[Title/Abstract])) OR (Mammary Cancer[Title/Abstract])) OR (Mammary Cancers[Title/Abstract])) OR (Malignant Neoplasm of Breast[Title/Abstract])) OR (Breast Malignant Neoplasm[Title/Abstract])) OR (Breast Malignant Neoplasms[Title/Abstract])) OR (Malignant Tumor of Breast[Title/Abstract])) OR (Breast Malignant Tumor[Title/Abstract])) OR (Breast Malignant Tumors[Title/Abstract])) OR (Cancer of Breast[Title/Abstract])) OR (Cancer of the Breast[Title/Abstract])) OR (Human Mammary Carcinomas[Title/Abstract])) OR (Human Mammary Carcinoma[Title/Abstract])) OR (Breast Carcinoma[Title/Abstract])) OR (Breast Carcinomas[Title/Abstract])) OR (breast tumor[Title/Abstract])) OR (breast gland tumor[Title/Abstract])) OR (breast gland tumour[Title/Abstract])) OR (breast mass[Title/Abstract])) OR (mamma tumor[Title/Abstract])) OR (mamma tumour[Title/Abstract])) OR (mammary gland neoplasia[Title/Abstract])) OR (mammary gland neoplasm[Title/Abstract])) OR (mammary gland tumor[Title/Abstract])) OR (mammary gland tumorigenesis[Title/Abstract])) OR (mammary neoplasia[Title/Abstract])) OR (mammary neoplasm[Title/Abstract])) OR (mammary neoplasms[Title/Abstract])) OR (mammary tumorigenesis[Title/Abstract])) OR (neoplasia of the breast[Title/Abstract])) OR (neoplasm of the breast[Title/Abstract])) OR (neoplasm of the mammary gland[Title/Abstract])) OR (neoplastic breast[Title/Abstract])) OR (neoplastic mammary[Title/Abstract])) OR (tumor of the breast[Title/Abstract])) OR (tumor of the mammary gland[Title/Abstract])) OR (tumorigenesis of the breast[Title/Abstract])) OR (tumorigenesis of the mammary gland[Title/Abstract]))) AND ((machine learning[MeSH Terms]) OR ((((((((((((((((((machine learning[Title/Abstract]) OR (Transfer Learning[Title/Abstract])) OR (Deep learning[Title/Abstract])) OR (Ensemble Learning[Title/Abstract])) OR (artificial intelligence[Title/Abstract])) OR (neural network[Title/Abstract])) OR (neural networks[Title/Abstract])) OR (K-Nearest Neighbor[Title/Abstract])) OR (CNN[Title/Abstract]))) OR (AlexNet[Title/Abstract])) OR (VGGNet[Title/Abstract])) OR (ResNet[Title/Abstract])) OR (GoogLeNet[Title/Abstract])) OR (Radiomics[Title/Abstract])) OR (Radiomic[Title/Abstract])) OR (radiogenomic[Title/Abstract])) OR (Texture[Title/Abstract])))) AND ((Lymphatic Metastasis[MeSH Terms]) OR ((((Lymphatic Metastasis[Title/Abstract]) OR (Lymphatic Metastases[Title/Abstract])) OR (Lymph Node Metastasis[Title/Abstract])) OR (Lymph Node Metastases[Title/Abstract]))) | 317 |

**2.Cochrane**

| Search number | Query | Results |
| --- | --- | --- |
| #1 | MeSH descriptor: [Breast Neoplasms] explode all trees | 20228 |
| #2 | (Breast Neoplasms):ti,ab,kw OR (Breast Neoplasm):ti,ab,kw OR (Breast Tumors):ti,ab,kw OR (Breast Tumor):ti,ab,kw OR (Breast Cancer):ti,ab,kw | 47624 |
| #3 | (Mammary Cancer):ti,ab,kw OR (Mammary Cancers):ti,ab,kw OR (Malignant Neoplasm of Breast):ti,ab,kw OR (Breast Malignant Neoplasm):ti,ab,kw OR (Breast Malignant Neoplasms):ti,ab,kw | 2733 |
| #4 | (Malignant Tumor of Breast):ti,ab,kw OR (Breast Malignant Tumor):ti,ab,kw OR (Breast Malignant Tumors):ti,ab,kw OR (Cancer of Breast):ti,ab,kw OR (Cancer of the Breast):ti,ab,kw | 43631 |
| #5 | (Human Mammary Carcinomas):ti,ab,kw OR (Human Mammary Carcinoma):ti,ab,kw OR (Breast Carcinoma):ti,ab,kw OR (Breast Carcinomas):ti,ab,kw OR (breast tumor):ti,ab,kw | 14362 |
| #6 | (breast gland tumor):ti,ab,kw OR (breast gland tumour):ti,ab,kw OR (breast mass):ti,ab,kw OR (mamma tumor):ti,ab,kw OR (mamma tumour):ti,ab,kw | 4359 |
| #7 | (mammary gland neoplasia):ti,ab,kw OR (mammary gland neoplasm):ti,ab,kw OR (mammary gland tumor):ti,ab,kw OR (mammary gland tumorigenesis):ti,ab,kw OR (mammary neoplasia):ti,ab,kw | 43 |
| #8 | (mammary neoplasm):ti,ab,kw OR (mammary neoplasms):ti,ab,kw OR (mammary tumorigenesis):ti,ab,kw OR (neoplasia of the breast):ti,ab,kw OR (neoplasm of the breast):ti,ab,kw | 7071 |
| #9 | (neoplasm of the mammary gland):ti,ab,kw OR (neoplastic breast):ti,ab,kw OR (neoplastic mammary):ti,ab,kw OR (tumor of the breast):ti,ab,kw OR (tumor of the mammary gland):ti,ab,kw | 11296 |
| #10 | (tumorigenesis of the breast):ti,ab,kw OR (tumorigenesis of the mammary gland):ti,ab,kw | 52 |
| #11 | #1 OR #2 OR #3 OR #4 OR #5 OR #6 OR #7 OR #8 OR #9 OR #10 | 49340 |
| #12 | MeSH descriptor: [Machine Learning] explode all trees | 983 |
| #13 | (machine learning):ti,ab,kw OR (Transfer Learning):ti,ab,kw OR (Deep learning):ti,ab,kw OR (Ensemble Learning):ti,ab,kw OR (artificial intelligence):ti,ab,kw | 7380 |
| #14 | (neural network):ti,ab,kw OR (neural networks):ti,ab,kw OR (K-Nearest Neighbor):ti,ab,kw OR (CNN):ti,ab,kw OR (AlexNet):ti,ab,kw | 3779 |
| #15 | (VGGNet):ti,ab,kw OR (ResNet):ti,ab,kw OR (GoogLeNet):ti,ab,kw OR (Radiomics):ti,ab,kw OR (Radiomic):ti,ab,kw | 800 |
| #16 | (radiogenomic):ti,ab,kw OR (Texture):ti,ab,kw | 2139 |
| #17 | #12 OR #13 OR #14 OR #15 OR #16 | 12627 |
| #18 | MeSH descriptor: [Lymphatic Metastasis] explode all trees | 2755 |
| #19 | (Lymphatic Metastasis):ti,ab,kw OR (Lymphatic Metastases):ti,ab,kw OR (Lymph Node Metastasis):ti,ab,kw OR (Lymph Node Metastases):ti,ab,kw | 6839 |
| #20 | #18 OR #19 | 6839 |
| #21 | #11 AND #17 AND #20 | 17 |

**3.Embase**

| Search number | Query | Results |
| --- | --- | --- |
| #1 | 'breast tumor'/exp | 708210 |
| #2 | 'breast neoplasms':ab,ti OR 'breast tumors':ab,ti OR 'breast tumor':ab,ti OR 'breast cancer':ab,ti OR 'mammary cancer':ab,ti OR 'mammary cancers':ab,ti OR 'malignant neoplasm of breast':ab,ti OR 'breast malignant neoplasm':ab,ti OR 'breast malignant neoplasms':ab,ti OR 'malignant tumor of breast':ab,ti OR 'breast malignant tumor':ab,ti OR 'breast malignant tumors':ab,ti OR 'cancer of breast':ab,ti OR 'cancer of the breast':ab,ti OR 'human mammary carcinomas':ab,ti OR 'human mammary carcinoma':ab,ti OR 'breast carcinomas':ab,ti OR 'breast carcinoma':ab,ti OR 'breast gland tumour':ab,ti OR 'breast mass':ab,ti OR 'mamma tumor':ab,ti OR 'mamma tumour':ab,ti OR 'mammary gland neoplasia':ab,ti OR 'mammary gland neoplasm':ab,ti OR 'mammary gland tumor':ab,ti OR 'mammary gland tumorigenesis':ab,ti OR 'mammary neoplasia':ab,ti OR 'mammary neoplasm':ab,ti OR 'mammary neoplasms':ab,ti OR 'mammary tumorigenesis':ab,ti OR 'neoplasia of the breast':ab,ti OR 'neoplasm of the breast':ab,ti OR 'neoplasm of the mammary gland':ab,ti OR 'neoplastic breast':ab,ti OR 'neoplastic mammary':ab,ti OR 'tumor of the breast':ab,ti OR 'tumor of the mammary gland':ab,ti OR 'tumorigenesis of the breast':ab,ti OR 'tumorigenesis of the mammary gland':ab,ti | 552425 |
| #3 | #89 OR #90 | 767769 |
| #4 | 'machine learning'/exp | 479106 |
| #5 | 'machine learning':ab,ti OR 'transfer learning':ab,ti OR 'deep learning':ab,ti OR 'ensemble learning':ab,ti OR 'artificial intelligence':ab,ti OR 'neural network':ab,ti OR 'neural networks':ab,ti OR 'k-nearest neighbor':ab,ti OR cnn:ab,ti OR alexnet:ab,ti OR resnet:ab,ti OR googlenet:ab,ti OR radiomics:ab,ti OR radiomic:ab,ti OR radiogenomic:ab,ti OR texture:ab,ti OR vggnet:ab,ti | 345789 |
| #6 | #93 OR #94 | 609707 |
| #7 | 'lymph node metastasis'/exp | 182615 |
| #8 | 'lymphatic metastasis':ab,ti OR 'lymphatic metastases':ab,ti OR 'lymph node metastasis':ab,ti OR 'lymph node metastases':ab,ti | 89762 |
| #9 | #96 OR #97 | 197866 |
| #10 | #92 AND #95 AND #98 | 635 |

**4.Web of science**

| Search number | Query | Results |
| --- | --- | --- |
| #1 | Breast Neoplasms (Topic) OR Breast Neoplasm (Topic) OR Breast Tumors (Topic) OR Breast Tumor (Topic) OR Breast Cancer (Topic) OR Mammary Cancer (Topic) OR Mammary Cancers (Topic) OR Malignant Neoplasm of Breast (Topic) OR Breast Malignant Neoplasm (Topic) OR Breast Malignant Neoplasms (Topic) OR Malignant Tumor of Breast (Topic) OR Breast Malignant Tumor (Topic) OR Breast Malignant Tumors (Topic) OR Cancer of Breast (Topic) OR Cancer of the Breast (Topic) OR Human Mammary Carcinomas (Topic) OR Human Mammary Carcinoma (Topic) OR Breast Carcinoma (Topic) OR Breast Carcinomas (Topic) OR breast tumor (Topic) OR breast gland tumor (Topic) OR breast gland tumour (Topic) OR breast mass (Topic) OR mamma tumor (Topic) OR mamma tumour (Topic) OR mammary gland neoplasia (Topic) OR mammary gland neoplasm (Topic) OR mammary gland tumor (Topic) OR mammary gland tumorigenesis (Topic) OR mammary neoplasia (Topic) OR mammary neoplasm (Topic) OR mammary neoplasms (Topic) OR mammary tumorigenesis (Topic) OR neoplasia of the breast (Topic) OR neoplasm of the breast (Topic) OR neoplasm of the mammary gland (Topic) OR neoplastic breast (Topic) OR neoplastic mammary (Topic) OR tumor of the breast (Topic) OR tumor of the mammary gland (Topic) OR tumorigenesis of the breast (Topic) OR tumorigenesis of the mammary gland (Topic) | 761271 |
| #2 | machine learning (Topic) OR Transfer Learning (Topic) OR Deep learning (Topic) OR Ensemble Learning (Topic) OR artificial intelligence (Topic) OR neural network (Topic) OR neural networks (Topic) OR K-Nearest Neighbor (Topic) OR CNN (Topic) OR AlexNet (Topic) OR VGGNet (Topic) OR ResNet (Topic) OR GoogLeNet (Topic) OR Radiomics (Topic) OR Radiomic (Topic) OR radiogenomic (Topic) OR Texture (Topic) | 1674587 |
| #3 | Lymphatic Metastasis (Topic) OR Lymphatic Metastases (Topic) OR Lymph Node Metastasis (Topic) OR Lymph Node Metastases (Topic) | 110862 |
| #4 | #1 AND #2 AND #3 | 446 |
